# Supplementary figures and images for: Proteomic profiling of urinary extracellular vesicles differentiates breast cancer patients from healthy women
Source: PLoS One. 2023 Nov 3;18(11):e0291574. doi: 10.1371/journal.pone.0291574 (PMC10624262; doi:10.1371/journal.pone.0291574)

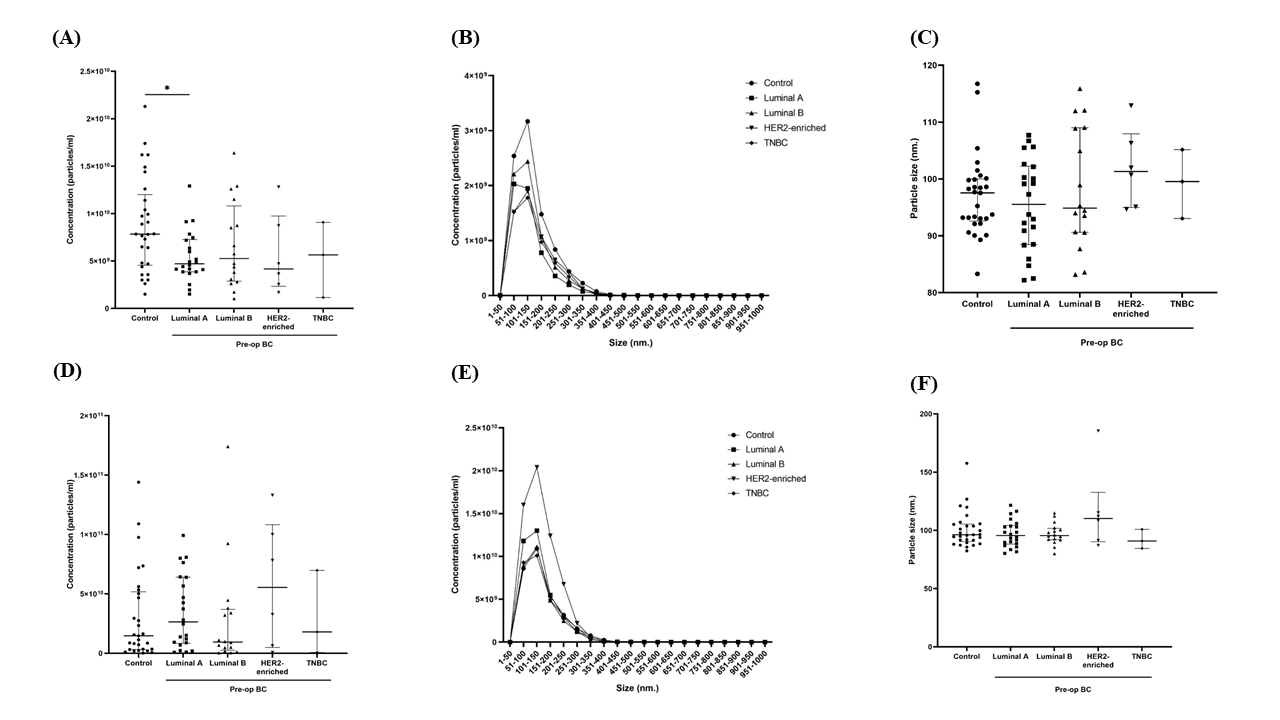

Supplement: S1 Fig — (A) Total particle concentration in urine (median ± IQR). (B) Total particle size distribution in urine. (C) Mode size of total particles in urine according to BC subtype (median ± IQR). (D) uEV concentration (median ± IQR). (E) uEV size distribution. (F) Mode size of uEVs according to BC subtype (median ± IQR). Results were derived from 29 CT samples and 19, 6, 6, and 3 samples from BC patients with luminal A, luminal B, HER2-enriched, and TNBC subtypes, respectively. *: p < 0.05. (TIF) [file pone.0291574.s001.tif]

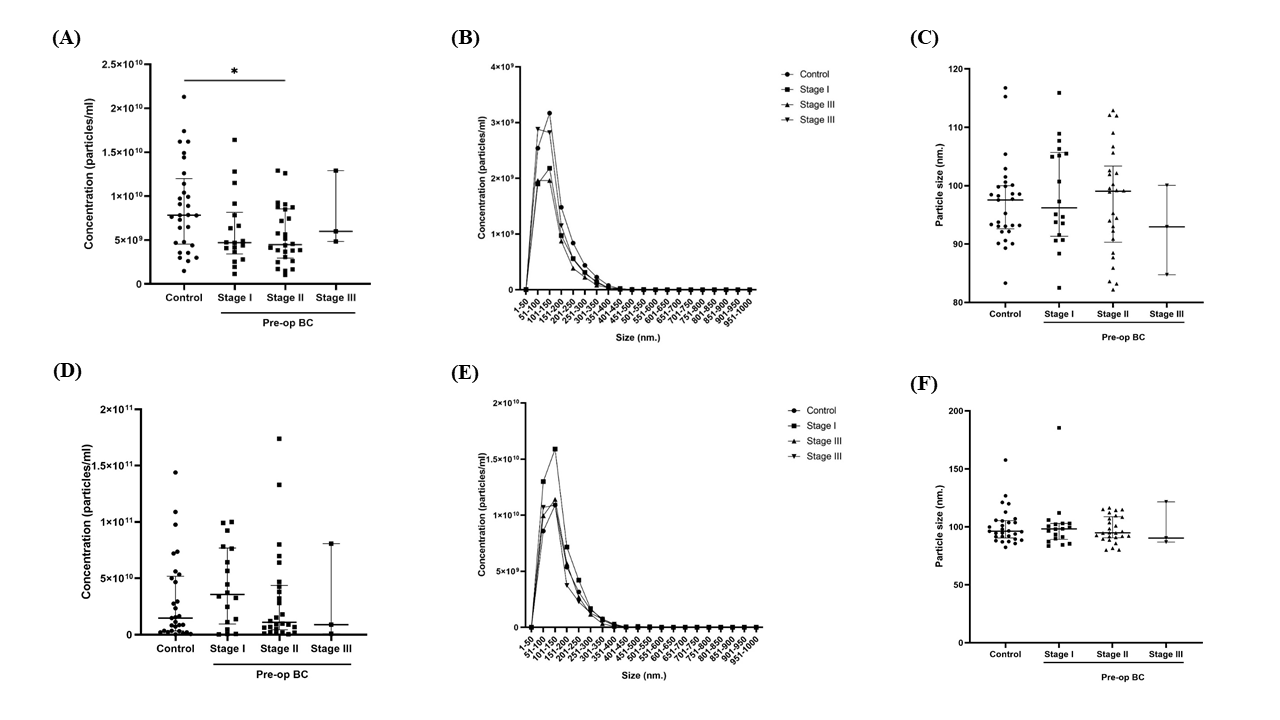

Supplement: S2 Fig — (A) Total particle concentration in urine (median ± IQR). (B) Total particle size distribution in urine. (C) Mode size of total particles in urine according to BC subtype (median ± IQR). (D) uEV concentration (median ± IQR). (E) uEV size distribution. (F) Mode size of uEVs according to BC subtype (median ± IQR). Results were derived from 29 CT samples and 19, 6, 6, and 3 samples from BC patients with luminal A, luminal B, HER2-enriched, and TNBC subtypes, respectively. *: p < 0.05. (TIF) [file pone.0291574.s002.tif]

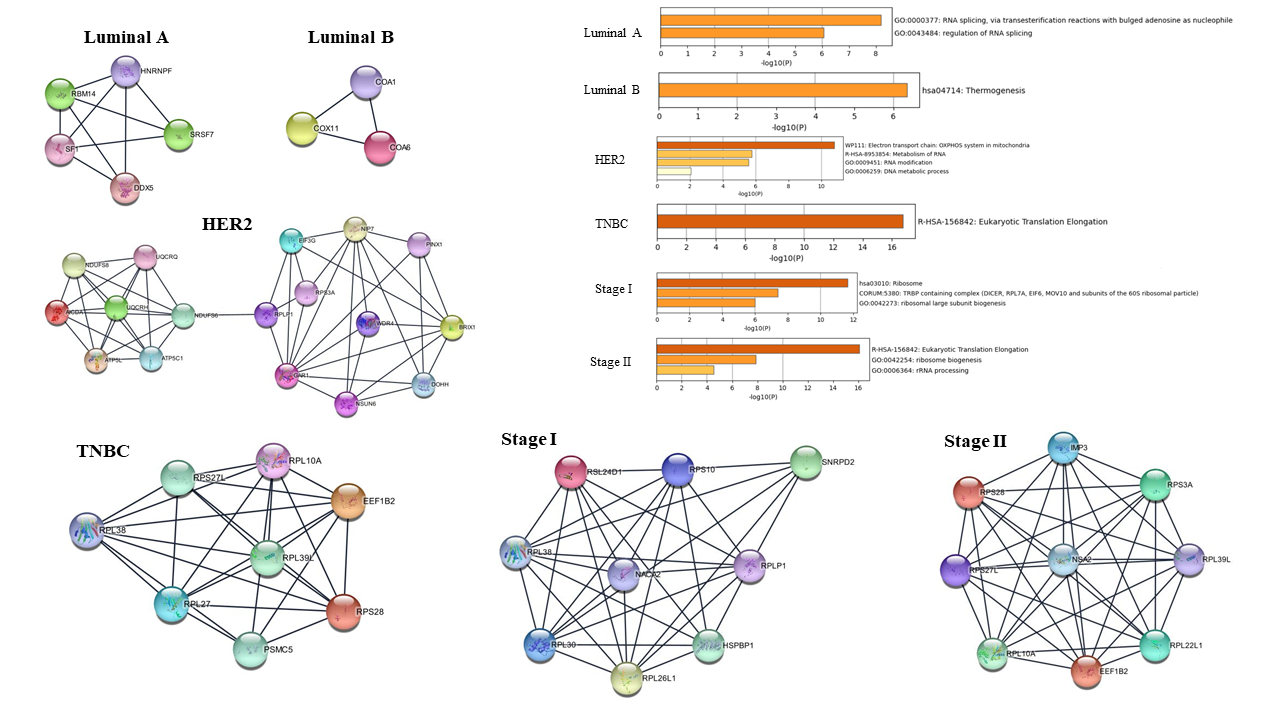

Supplement: S3 Fig — The hub proteins were selected from the protein-protein interaction network annotated by STRING operated by the MCODE algorithm using Cytoscape. The cutoff criteria were set as follows: degree cutoff = 2, node score cutoff = 0.2, k-core = 2, and maximum depth = 100. (TIF) [file pone.0291574.s003.tif]

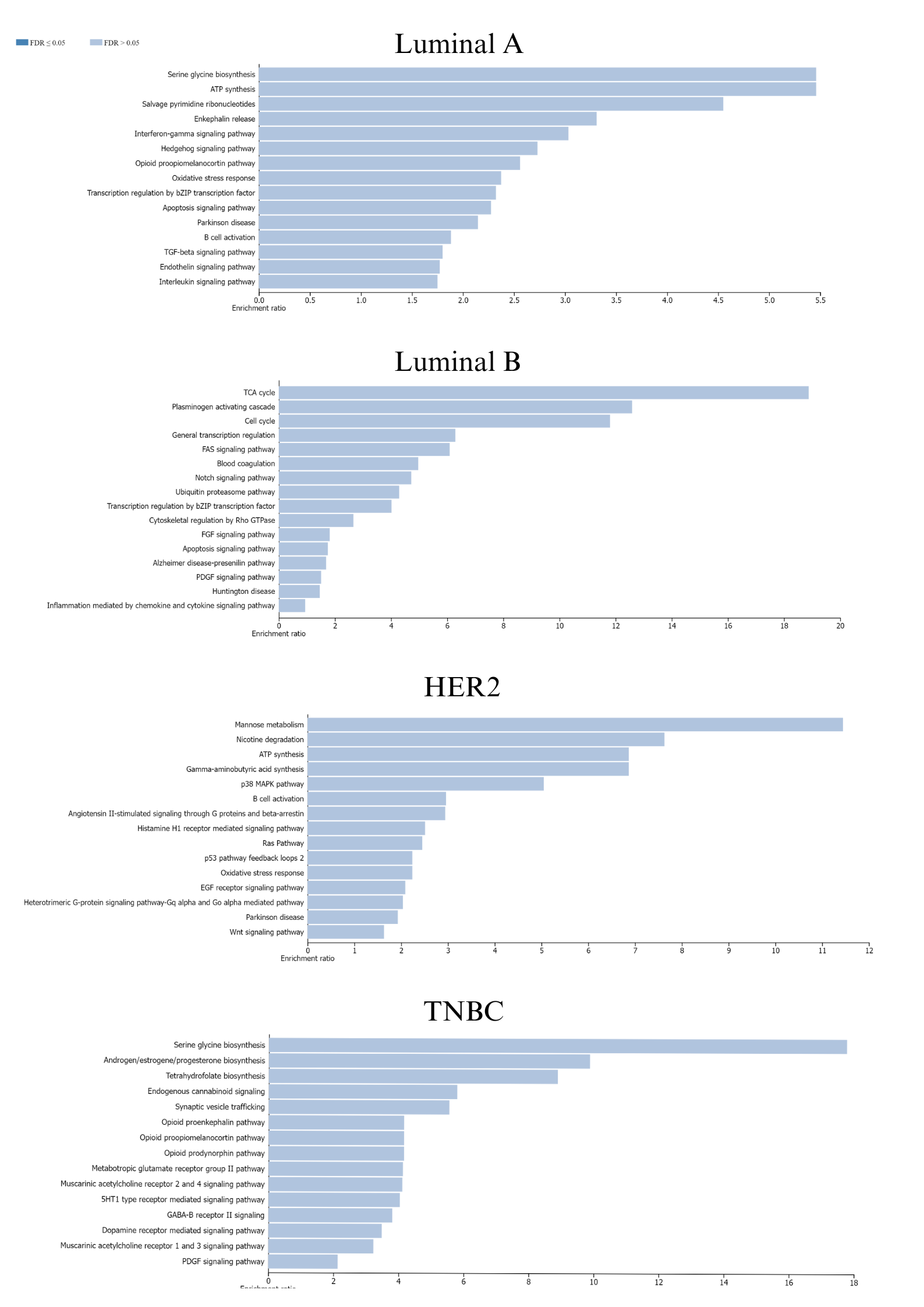

Supplement: S4 Fig — The top 15 pathways were determined using WEB-based GEne SeT AnaLysis Toolkit (WebGestalt). (TIF) [file pone.0291574.s004.tif]

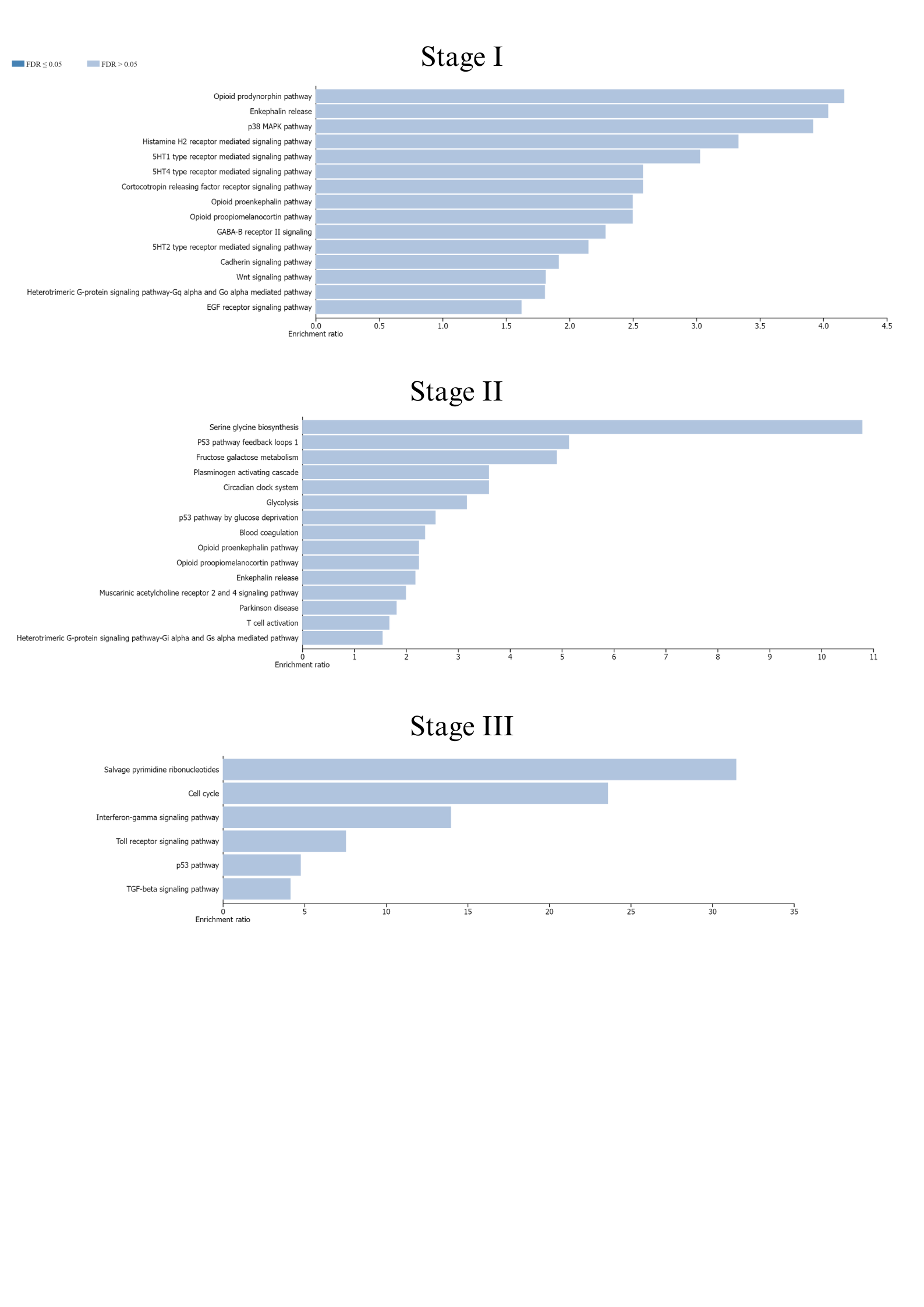

Supplement: S5 Fig — The top 15 pathways were determined using WEB-based GEne SeT AnaLysis Toolkit (WebGestalt). (TIF) [file pone.0291574.s005.tif]
