# Supplementary material for: Proteomic profiling of urinary extracellular vesicles differentiates breast cancer patients from healthy women
Source: PLoS One. 2023 Nov 3;18(11):e0291574. doi: 10.1371/journal.pone.0291574 (PMC10624262; doi:10.1371/journal.pone.0291574)
Supplement: S1 Table — (DOCX) [file pone.0291574.s006.docx]

**S1 Table. The 259 significantly DEPs (FC≥4) in uEVs of BC compared with CT.**

| **Entry** | **Fold change (FC)** | **log2(FC)** | **Adjusted**  **p-value** |
| --- | --- | --- | --- |
| TAU_HUMAN | 187.96 | 7.5543 | 0.038369 |
| DHH_HUMAN | 149.26 | 7.2217 | 0.01331 |
| CRBG1_HUMAN | 133.97 | 7.0658 | 0.00162 |
| CAC1C_HUMAN | 96.726 | 6.5958 | 0.029592 |
| KCNB2_HUMAN | 78.387 | 6.2925 | 0.018222 |
| LMLN_HUMAN | 76.374 | 6.255 | 0.004148 |
| RB39A_HUMAN | 76.033 | 6.2486 | 0.004428 |
| SPAG5_HUMAN | 72.956 | 6.189 | 0.005973 |
| KDM1B_HUMAN | 71.11 | 6.152 | 0.000661 |
| P66B_HUMAN | 69.095 | 6.1105 | 0.017297 |
| DOT1L_HUMAN | 59.763 | 5.9012 | 0.043167 |
| RAB7B_HUMAN | 59.396 | 5.8923 | 0.002043 |
| CLCKA_HUMAN | 46.806 | 5.5486 | 0.011375 |
| SHAN2_HUMAN | 42.214 | 5.3996 | 0.015142 |
| PHLB1_HUMAN | 40.231 | 5.3302 | 0.014977 |
| FEM1A_HUMAN | 37.85 | 5.2422 | 0.012864 |
| DAPLE_HUMAN | 35.473 | 5.1486 | 0.011783 |
| IRAK4_HUMAN | 34.159 | 5.0942 | 0.040095 |
| MY15B_HUMAN | 32.86 | 5.0383 | 0.035779 |
| KI67_HUMAN | 32.836 | 5.0372 | 0.002766 |
| H18_HUMAN | 32.467 | 5.0209 | 0.00223 |
| DYH1_HUMAN | 29.839 | 4.8991 | 0.009974 |
| RBM6_HUMAN | 28.563 | 4.8361 | 0.045936 |
| SMG1_HUMAN | 26.675 | 4.7374 | 0.032018 |
| RGS14_HUMAN | 26.378 | 4.7213 | 0.046996 |
| CWC22_HUMAN | 26.065 | 4.7041 | 0.018288 |
| ARI1B_HUMAN | 24.254 | 4.6002 | 0.007745 |
| GRTP1_HUMAN | 23.04 | 4.526 | 0.001741 |
| BPIB6_HUMAN | 22.664 | 4.5023 | 0.004212 |
| GRM5_HUMAN | 21.837 | 4.4487 | 0.005983 |
| SRGP2_HUMAN | 21.431 | 4.4216 | 0.002103 |
| ATAD2_HUMAN | 20.708 | 4.3721 | 0.00042 |
| DLG3_HUMAN | 20.7 | 4.3716 | 0.008416 |
| K0825_HUMAN | 20.157 | 4.3332 | 0.022513 |
| POSTN_HUMAN | 20.114 | 4.3301 | 0.02424 |
| ZN148_HUMAN | 19.411 | 4.2788 | 0.00042 |
| KCNU1_HUMAN | 19.031 | 4.2503 | 0.029457 |
| ARTN_HUMAN | 19.011 | 4.2488 | 0.017251 |
| SHLD3_HUMAN | 17.943 | 4.1653 | 0.011783 |
| ZN541_HUMAN | 17.785 | 4.1526 | 0.021226 |
| TUTLA_HUMAN | 17.682 | 4.1442 | 0.00223 |
| ASPN_HUMAN | 17.274 | 4.1105 | 0.033124 |
| TIE2_HUMAN | 16.472 | 4.042 | 0.010145 |
| FGD6_HUMAN | 16.384 | 4.0342 | 0.004927 |
| O10P1_HUMAN | 15.417 | 3.9464 | 0.000456 |
| FERM1_HUMAN | 15.21 | 3.9269 | 0.003458 |
| KDM4A_HUMAN | 14.81 | 3.8885 | 0.033818 |
| RN123_HUMAN | 14.48 | 3.856 | 0.014218 |
| BTBDH_HUMAN | 13.866 | 3.7934 | 0.016377 |
| ZBT49_HUMAN | 13.811 | 3.7877 | 0.013091 |
| B3GN2_HUMAN | 13.745 | 3.7809 | 0.000714 |
| FKB15_HUMAN | 13.62 | 3.7677 | 0.033092 |
| TYK2_HUMAN | 13.513 | 3.7563 | 0.005064 |
| PODN_HUMAN | 12.83 | 3.6815 | 0.035196 |
| TCF15_HUMAN | 12.805 | 3.6786 | 0.006499 |
| MED24_HUMAN | 12.658 | 3.662 | 0.001434 |
| RBP1_HUMAN | 12.556 | 3.6503 | 0.046121 |
| BRD9_HUMAN | 12.502 | 3.6441 | 0.014202 |
| GSK3B_HUMAN | 12.483 | 3.6419 | 0.00468 |
| NRM_HUMAN | 12.27 | 3.617 | 0.000177 |
| HRES1_HUMAN | 12.039 | 3.5897 | 0.023384 |
| UNC80_HUMAN | 11.192 | 3.4844 | 0.004957 |
| ICAL_HUMAN | 11.111 | 3.474 | 0.045522 |
| LTBP4_HUMAN | 11.082 | 3.4702 | 0.032682 |
| TMCC3_HUMAN | 10.952 | 3.4532 | 0.004617 |
| TERA_HUMAN | 10.418 | 3.3811 | 0.022594 |
| RPF1_HUMAN | 10.364 | 3.3736 | 0.012864 |
| MDM4_HUMAN | 10.348 | 3.3713 | 0.00338 |
| FYCO1_HUMAN | 10.119 | 3.339 | 0.000889 |
| ZN800_HUMAN | 9.6765 | 3.2745 | 0.024087 |
| T131L_HUMAN | 9.4938 | 3.247 | 0.007298 |
| HXK1_HUMAN | 9.3514 | 3.2252 | 0.00039 |
| MTEF4_HUMAN | 9.2987 | 3.217 | 0.025827 |
| CNGB3_HUMAN | 8.961 | 3.1637 | 0.022513 |
| SH3L2_HUMAN | 8.946 | 3.1612 | 0.011783 |
| EMAL5_HUMAN | 8.2861 | 3.0507 | 0.032691 |
| TNR12_HUMAN | 8.1379 | 3.0246 | 0.002103 |
| NUMA1_HUMAN | 7.9928 | 2.9987 | 0.014499 |
| P4K2B_HUMAN | 7.9836 | 2.997 | 0.024087 |
| OBSCN_HUMAN | 7.8969 | 2.9813 | 0.02057 |
| UBP44_HUMAN | 7.8434 | 2.9715 | 0.011783 |
| BAZ2B_HUMAN | 7.6518 | 2.9358 | 0.001353 |
| DDX28_HUMAN | 7.5943 | 2.9249 | 0.015884 |
| MOONR_HUMAN | 7.5798 | 2.9222 | 0.018203 |
| GAB1_HUMAN | 7.497 | 2.9063 | 0.048791 |
| PABP3_HUMAN | 7.4941 | 2.9058 | 0.00662 |
| FAK2_HUMAN | 7.4699 | 2.9011 | 0.000456 |
| DMP1_HUMAN | 7.427 | 2.8928 | 0.020786 |
| MLH3_HUMAN | 7.3803 | 2.8837 | 0.019092 |
| ZNF34_HUMAN | 7.3528 | 2.8783 | 0.004212 |
| PIN4_HUMAN | 7.2658 | 2.8611 | 0.001765 |
| PA2GX_HUMAN | 7.2416 | 2.8563 | 0.014335 |
| GP160_HUMAN | 7.2277 | 2.8535 | 0.034733 |
| TIAM1_HUMAN | 7.1249 | 2.8329 | 0.007625 |
| WDR87_HUMAN | 7.0872 | 2.8252 | 0.001093 |
| ITAE_HUMAN | 7.0342 | 2.8144 | 0.02538 |
| RL22_HUMAN | 7.0109 | 2.8096 | 0.016576 |
| TTC27_HUMAN | 7.01 | 2.8094 | 0.009159 |
| CFA46_HUMAN | 6.9069 | 2.788 | 0.011923 |
| HHIP_HUMAN | 6.9024 | 2.7871 | 0.005619 |
| FGD4_HUMAN | 6.8761 | 2.7816 | 0.016077 |
| PLEC_HUMAN | 6.8654 | 2.7794 | 0.014247 |
| LFNG_HUMAN | 6.6863 | 2.7412 | 0.001353 |
| AHNK_HUMAN | 6.6135 | 2.7254 | 0.044928 |
| BTG4_HUMAN | 6.6059 | 2.7238 | 0.034324 |
| BCAS4_HUMAN | 6.5886 | 2.72 | 0.004098 |
| DC1L1_HUMAN | 6.5728 | 2.7165 | 0.019367 |
| PG12A_HUMAN | 6.4384 | 2.6867 | 0.009471 |
| SREC_HUMAN | 6.3257 | 2.6612 | 0.016162 |
| ZN462_HUMAN | 6.3193 | 2.6598 | 0.001353 |
| S26A7_HUMAN | 6.2119 | 2.635 | 0.016077 |
| LIPB1_HUMAN | 6.2089 | 2.6343 | 0.005335 |
| PPB1_HUMAN | 6.084 | 2.605 | 0.006063 |
| PCID2_HUMAN | 6.0762 | 2.6032 | 0.016623 |
| KCNQ3_HUMAN | 5.9714 | 2.5781 | 0.017712 |
| BRE1A_HUMAN | 5.9493 | 2.5727 | 0.010013 |
| VPS50_HUMAN | 5.9032 | 2.5615 | 0.028425 |
| TBX18_HUMAN | 5.8893 | 2.5581 | 0.032682 |
| HROB_HUMAN | 5.7667 | 2.5277 | 0.045994 |
| SDA1_HUMAN | 5.5543 | 2.4736 | 0.049293 |
| CP21A_HUMAN | 5.3816 | 2.428 | 0.038369 |
| BAZ1B_HUMAN | 5.3168 | 2.4106 | 0.04804 |
| DCAM_HUMAN | 5.1949 | 2.3771 | 0.014607 |
| ARI4A_HUMAN | 5.1867 | 2.3748 | 0.000858 |
| OXLA_HUMAN | 5.1707 | 2.3704 | 0.02047 |
| I17RE_HUMAN | 5.1357 | 2.3606 | 0.017775 |
| CIART_HUMAN | 5.1349 | 2.3603 | 0.003465 |
| DAAF5_HUMAN | 5.1282 | 2.3585 | 0.007298 |
| MFN2_HUMAN | 5.1182 | 2.3556 | 0.034324 |
| ARG33_HUMAN | 5.0833 | 2.3458 | 0.002422 |
| TP8L1_HUMAN | 5.0689 | 2.3417 | 0.004547 |
| GLT17_HUMAN | 5.0106 | 2.325 | 0.020021 |
| AT2C2_HUMAN | 4.9975 | 2.3212 | 0.005338 |
| SWT1_HUMAN | 4.9322 | 2.3022 | 0.012541 |
| NELL1_HUMAN | 4.8272 | 2.2712 | 0.004357 |
| ATMIN_HUMAN | 4.765 | 2.2525 | 0.001736 |
| DGKK_HUMAN | 4.755 | 2.2494 | 0.016626 |
| IPIL2_HUMAN | 4.576 | 2.1941 | 0.030411 |
| PAXB1_HUMAN | 4.5093 | 2.1729 | 0.004172 |
| MCU_HUMAN | 4.4818 | 2.1641 | 0.005179 |
| FBX33_HUMAN | 4.4517 | 2.1544 | 0.036483 |
| Z512B_HUMAN | 4.4374 | 2.1497 | 0.014511 |
| RBP2_HUMAN | 4.369 | 2.1273 | 0.033124 |
| FHOD3_HUMAN | 4.3626 | 2.1252 | 0.001893 |
| SEPP1_HUMAN | 4.3451 | 2.1194 | 0.028792 |
| KRA62_HUMAN | 4.31 | 2.1077 | 0.014271 |
| COQ8B_HUMAN | 4.3049 | 2.106 | 0.022513 |
| ACTN1_HUMAN | 4.2969 | 2.1033 | 0.01101 |
| MED1_HUMAN | 4.2786 | 2.0971 | 0.023384 |
| HKDC1_HUMAN | 4.2376 | 2.0833 | 0.015953 |
| D19L1_HUMAN | 4.226 | 2.0793 | 0.035779 |
| RIN3_HUMAN | 4.1588 | 2.0562 | 0.011188 |
| PTN4_HUMAN | 4.0894 | 2.0319 | 0.013653 |
| ZSWM8_HUMAN | 4.0576 | 2.0206 | 0.032691 |
| LCA5_HUMAN | 4.0114 | 2.0041 | 0.002758 |
| SHH_HUMAN | 0.24969 | -2.0018 | 0.002639 |
| MED26_HUMAN | 0.24803 | -2.0114 | 0.00731 |
| SOX4_HUMAN | 0.24786 | -2.0124 | 0.001353 |
| O52L2_HUMAN | 0.24776 | -2.013 | 0.00392 |
| CC086_HUMAN | 0.24764 | -2.0137 | 0.006679 |
| YD021_HUMAN | 0.24572 | -2.0249 | 0.001077 |
| TFKLJ_HUMAN | 0.24509 | -2.0286 | 0.000456 |
| NUP98_HUMAN | 0.24318 | -2.0399 | 3.23E-06 |
| KS6B2_HUMAN | 0.24302 | -2.0408 | 0.00589 |
| TASO2_HUMAN | 0.24184 | -2.0479 | 0.004357 |
| O11G2_HUMAN | 0.24152 | -2.0498 | 0.004565 |
| HDAC6_HUMAN | 0.24054 | -2.0556 | 0.001353 |
| LORF2_HUMAN | 0.23641 | -2.0806 | 1.61E-09 |
| RBG1L_HUMAN | 0.23555 | -2.0859 | 0.013887 |
| NBEA_HUMAN | 0.233 | -2.1016 | 9.79E-05 |
| CD158_HUMAN | 0.23166 | -2.1099 | 0.009924 |
| MA7D1_HUMAN | 0.23086 | -2.1149 | 0.000976 |
| TMCO6_HUMAN | 0.23019 | -2.1191 | 0.034104 |
| MA7D2_HUMAN | 0.23007 | -2.1199 | 0.000891 |
| P4HA1_HUMAN | 0.22879 | -2.1279 | 0.000156 |
| CF298_HUMAN | 0.2267 | -2.1411 | 4.26E-05 |
| ZN500_HUMAN | 0.22627 | -2.1439 | 0.007625 |
| TSR1_HUMAN | 0.22605 | -2.1453 | 0.016377 |
| SP14L_HUMAN | 0.22558 | -2.1483 | 0.000933 |
| ANR17_HUMAN | 0.22377 | -2.1599 | 0.000969 |
| AP2B1_HUMAN | 0.22376 | -2.16 | 0.001344 |
| SPHM_HUMAN | 0.2223 | -2.1694 | 0.000551 |
| RB12B_HUMAN | 0.22001 | -2.1843 | 0.01331 |
| STX19_HUMAN | 0.21996 | -2.1847 | 0.003974 |
| NFH_HUMAN | 0.21981 | -2.1857 | 0.016204 |
| OVOL2_HUMAN | 0.21882 | -2.1922 | 0.000446 |
| IRPL2_HUMAN | 0.2186 | -2.1937 | 6.51E-05 |
| CK087_HUMAN | 0.21381 | -2.2256 | 9.35E-07 |
| NR2F6_HUMAN | 0.21332 | -2.2289 | 0.000969 |
| RPC4_HUMAN | 0.2103 | -2.2495 | 0.00018 |
| SIDT1_HUMAN | 0.21005 | -2.2512 | 3.25E-07 |
| MEST_HUMAN | 0.20939 | -2.2557 | 0.00042 |
| TCTP8_HUMAN | 0.20742 | -2.2693 | 4.09E-05 |
| ZCH13_HUMAN | 0.20355 | -2.2966 | 0.000667 |
| FA81A_HUMAN | 0.20247 | -2.3042 | 1.54E-05 |
| DHX15_HUMAN | 0.20098 | -2.3149 | 0.023647 |
| CBPM_HUMAN | 0.20061 | -2.3176 | 5.63E-05 |
| LAP4B_HUMAN | 0.19612 | -2.3502 | 0.013091 |
| K1C28_HUMAN | 0.19542 | -2.3553 | 0.00731 |
| HAUS4_HUMAN | 0.19521 | -2.3569 | 0.002008 |
| LRC28_HUMAN | 0.19514 | -2.3574 | 0.001452 |
| PLCE1_HUMAN | 0.19351 | -2.3695 | 3.25E-07 |
| MSTRO_HUMAN | 0.18876 | -2.4054 | 0.034124 |
| OTULL_HUMAN | 0.18842 | -2.408 | 0.007298 |
| GABP2_HUMAN | 0.18323 | -2.4482 | 0.025659 |
| NADAP_HUMAN | 0.18097 | -2.4662 | 0.003558 |
| ATG9B_HUMAN | 0.1808 | -2.4676 | 0.000204 |
| WDR76_HUMAN | 0.1801 | -2.4731 | 0.002724 |
| GRB1L_HUMAN | 0.17987 | -2.475 | 0.000805 |
| SYQ_HUMAN | 0.17655 | -2.5018 | 0.014944 |
| CAPS2_HUMAN | 0.17386 | -2.524 | 0.000408 |
| MPP3_HUMAN | 0.16815 | -2.5722 | 0.000392 |
| TCF20_HUMAN | 0.16746 | -2.5781 | 6.32E-05 |
| PGM1_HUMAN | 0.16576 | -2.5929 | 0.00162 |
| CP27B_HUMAN | 0.16574 | -2.593 | 0.000204 |
| NF1_HUMAN | 0.16533 | -2.5966 | 0.006376 |
| CIPC_HUMAN | 0.16375 | -2.6104 | 0.000383 |
| ZN580_HUMAN | 0.15976 | -2.646 | 0.000137 |
| PAGE1_HUMAN | 0.15932 | -2.65 | 0.014944 |
| WFS1_HUMAN | 0.1562 | -2.6785 | 0.000858 |
| AK1BA_HUMAN | 0.15591 | -2.6812 | 0.004691 |
| INCE_HUMAN | 0.15478 | -2.6917 | 0.016313 |
| TFIP8_HUMAN | 0.15353 | -2.7034 | 0.001344 |
| INO80_HUMAN | 0.15332 | -2.7054 | 0.027949 |
| TTC12_HUMAN | 0.15228 | -2.7152 | 1.61E-09 |
| KRIT1_HUMAN | 0.15158 | -2.7219 | 9.25E-05 |
| CCNI2_HUMAN | 0.15026 | -2.7344 | 3.94E-09 |
| DB135_HUMAN | 0.14323 | -2.8036 | 0.00042 |
| ZN768_HUMAN | 0.13609 | -2.8774 | 0.011783 |
| SDK2_HUMAN | 0.13375 | -2.9024 | 0.000723 |
| RHG18_HUMAN | 0.13217 | -2.9195 | 0.006435 |
| BGAL_HUMAN | 0.12968 | -2.9469 | 9.75E-05 |
| AKP13_HUMAN | 0.12822 | -2.9633 | 0.00031 |
| FRM4A_HUMAN | 0.12809 | -2.9647 | 9.25E-05 |
| ACM4_HUMAN | 0.12172 | -3.0383 | 0.000889 |
| MARK1_HUMAN | 0.11857 | -3.0762 | 0.00162 |
| GRASP_HUMAN | 0.11822 | -3.0804 | 1.11E-06 |
| SPEF2_HUMAN | 0.1174 | -3.0905 | 0.00045 |
| SGK2_HUMAN | 0.1136 | -3.1379 | 4.60E-06 |
| ATL1_HUMAN | 0.11152 | -3.1646 | 5.05E-07 |
| TBX2_HUMAN | 0.11104 | -3.1708 | 0.0009 |
| 3MG_HUMAN | 0.11044 | -3.1786 | 0.000684 |
| OR2B6_HUMAN | 0.11014 | -3.1825 | 0.001466 |
| GALD1_HUMAN | 0.10969 | -3.1885 | 6.52E-06 |
| IPO11_HUMAN | 0.10238 | -3.2879 | 0.00039 |
| RENBP_HUMAN | 0.1016 | -3.2991 | 0.044622 |
| CK040_HUMAN | 0.09159 | -3.4487 | 0.037887 |
| TAAR5_HUMAN | 0.087862 | -3.5086 | 0.00042 |
| PCDAB_HUMAN | 0.080251 | -3.6393 | 0.001621 |
| GG6L7_HUMAN | 0.077574 | -3.6883 | 0.00026 |
| SURF6_HUMAN | 0.073864 | -3.759 | 0.000546 |
| CLCN7_HUMAN | 0.073859 | -3.7591 | 0.005339 |
| TRPM8_HUMAN | 0.073672 | -3.7627 | 4.60E-06 |
| CAC1I_HUMAN | 0.063418 | -3.979 | 0.046769 |
| SO1B1_HUMAN | 0.059812 | -4.0634 | 0.001557 |
| YJU2_HUMAN | 0.051859 | -4.2693 | 1.56E-05 |
| LYL1_HUMAN | 0.041554 | -4.5889 | 1.84E-05 |
| NET4_HUMAN | 0.022458 | -5.4766 | 0.000857 |
| EST1_HUMAN | 0.004291 | -7.8646 | 0.000504 |
