# Supplementary material for: Proteomic profiling of urinary extracellular vesicles differentiates breast cancer patients from healthy women
Source: PLoS One. 2023 Nov 3;18(11):e0291574. doi: 10.1371/journal.pone.0291574 (PMC10624262; doi:10.1371/journal.pone.0291574)
Supplement: S2 Table — (DOCX) [file pone.0291574.s007.docx]

**S2 Table. PANTHER pathway analysis of the up-regulated DEPs (FC≥4) in uEVs of compared with CT.**

| **No.** | **PANTHER pathways** | **Genes** | **Percent of gene hit against total # genes** | **Percent of gene hit against total # Pathway hits** |
| --- | --- | --- | --- | --- |
| 1 | Beta2 adrenergic receptor signaling pathway (P04378) | 1 | 0.70% | 1.70% |
| 2 | Metabotropic glutamate receptor group III pathway (P00039) | 1 | 0.70% | 1.70% |
| 3 | Beta1 adrenergic receptor signaling pathway (P04377) | 1 | 0.70% | 1.70% |
| 4 | Angiogenesis (P00005) | 2 | 1.30% | 3.40% |
| 5 | Ionotropic glutamate receptor pathway (P00037) | 1 | 0.70% | 1.70% |
| 6 | Alzheimer disease-presenilin pathway (P00004) | 1 | 0.70% | 1.70% |
| 7 | Interleukin signaling pathway (P00036) | 1 | 0.70% | 1.70% |
| 8 | 5HT2 type receptor mediated signaling pathway (P04374) | 1 | 0.70% | 1.70% |
| 9 | Alzheimer disease-amyloid secretase pathway (P00003) | 1 | 0.70% | 1.70% |
| 10 | Integrin signalling pathway (P00034) | 3 | 2.00% | 5.10% |
| 11 | 5-Hydroxytryptamine degredation (P04372) | 1 | 0.70% | 1.70% |
| 12 | Insulin/IGF pathway-protein kinase B signaling cascade (P00033) | 2 | 1.30% | 3.40% |
| 13 | Inflammation mediated by chemokine and cytokine signaling pathway (P00031) | 3 | 2.00% | 5.10% |
| 14 | Pentose phosphate pathway (P02762) | 2 | 1.30% | 3.40% |
| 15 | Nicotine pharmacodynamics pathway (P06587) | 1 | 0.70% | 1.70% |
| 16 | Huntington disease (P00029) | 2 | 1.30% | 3.40% |
| 17 | Endogenous cannabinoid signaling (P05730) | 1 | 0.70% | 1.70% |
| 18 | Heterotrimeric G-protein signaling pathway-rod outer segment phototransduction (P00028) | 1 | 0.70% | 1.70% |
| 19 | p53 pathway (P00059) | 1 | 0.70% | 1.70% |
| 20 | p53 pathway feedback loops 2 (P04398) | 1 | 0.70% | 1.70% |
| 21 | Heterotrimeric G-protein signaling pathway-Gq alpha and Go alpha mediated pathway (P00027) | 2 | 1.30% | 3.40% |
| 22 | Heterotrimeric G-protein signaling pathway-Gi alpha and Gs alpha mediated pathway (P00026) | 3 | 2.00% | 5.10% |
| 23 | Wnt signaling pathway (P00057) | 2 | 1.30% | 3.40% |
| 24 | Hedgehog signaling pathway (P00025) | 1 | 0.70% | 1.70% |
| 25 | Glycolysis (P00024) | 2 | 1.30% | 3.40% |
| 26 | Toll receptor signaling pathway (P00054) | 1 | 0.70% | 1.70% |
| 27 | Ras Pathway (P04393) | 2 | 1.30% | 3.40% |
| 28 | P53 pathway feedback loops 1 (P04392) | 1 | 0.70% | 1.70% |
| 29 | Oxytocin receptor mediated signaling pathway (P04391) | 1 | 0.70% | 1.70% |
| 30 | EGF receptor signaling pathway (P00018) | 1 | 0.70% | 1.70% |
| 31 | PI3 kinase pathway (P00048) | 1 | 0.70% | 1.70% |
| 32 | PDGF signaling pathway (P00047) | 3 | 2.00% | 5.10% |
| 33 | Notch signaling pathway (P00045) | 1 | 0.70% | 1.70% |
| 34 | Cadherin signaling pathway (P00012) | 1 | 0.70% | 1.70% |
| 35 | Nicotinic acetylcholine receptor signaling pathway (P00044) | 1 | 0.70% | 1.70% |
| 36 | Fructose galactose metabolism (P02744) | 2 | 1.30% | 3.40% |
| 37 | Metabotropic glutamate receptor group I pathway (P00041) | 1 | 0.70% | 1.70% |
| 38 | CCKR signaling map (P06959) | 2 | 1.30% | 3.40% |
| 39 | Gonadotropin-releasing hormone receptor pathway (P06664) | 3 | 2.00% | 5.10% |
