# Supplementary material for: Proteomic profiling of urinary extracellular vesicles differentiates breast cancer patients from healthy women
Source: PLoS One. 2023 Nov 3;18(11):e0291574. doi: 10.1371/journal.pone.0291574 (PMC10624262; doi:10.1371/journal.pone.0291574)
Supplement: S3 Table — (DOCX) [file pone.0291574.s008.docx]

**S3 Table. PANTHER pathway analysis of the down-regulated DEPs (FC≥4) in uEVs of compared with CT.**

| **No.** | **PANTHER pathways** | **Genes** | **Percent of gene hit against total # genes** | **Percent of gene hit against total # Pathway hits** |
| --- | --- | --- | --- | --- |
| 1 | Axon guidance mediated by netrin (P00009) | 1 | 1.00% | 3.60% |
| 2 | Axon guidance mediated by Slit/Robo (P00008) | 1 | 1.00% | 3.60% |
| 3 | Ionotropic glutamate receptor pathway (P00037) | 1 | 1.00% | 3.60% |
| 4 | Histamine H1 receptor mediated signaling pathway (P04385) | 1 | 1.00% | 3.60% |
| 5 | Alzheimer disease-amyloid secretase pathway (P00003) | 1 | 1.00% | 3.60% |
| 6 | Integrin signalling pathway (P00034) | 1 | 1.00% | 3.60% |
| 7 | Alpha adrenergic receptor signaling pathway (P00002) | 1 | 1.00% | 3.60% |
| 8 | Insulin/IGF pathway-mitogen activated protein kinase kinase/MAP kinase cascade (P00032) | 1 | 1.00% | 3.60% |
| 9 | Inflammation mediated by chemokine and cytokine signaling pathway (P00031) | 1 | 1.00% | 3.60% |
| 10 | p53 pathway by glucose deprivation (P04397) | 1 | 1.00% | 3.60% |
| 11 | EGF receptor signaling pathway (P00018) | 1 | 1.00% | 3.60% |
| 12 | Vitamin D metabolism and pathway (P04396) | 1 | 1.00% | 3.60% |
| 13 | PI3 kinase pathway (P00048) | 1 | 1.00% | 3.60% |
| 14 | PDGF signaling pathway (P00047) | 1 | 1.00% | 3.60% |
| 15 | Thyrotropin-releasing hormone receptor signaling pathway (P04394) | 1 | 1.00% | 3.60% |
| 16 | Nicotinic acetylcholine receptor signaling pathway (P00044) | 1 | 1.00% | 3.60% |
| 17 | Oxytocin receptor mediated signaling pathway (P04391) | 1 | 1.00% | 3.60% |
| 18 | Muscarinic acetylcholine receptor 2 and 4 signaling pathway (P00043) | 2 | 2.00% | 7.10% |
| 19 | Cadherin signaling pathway (P00012) | 1 | 1.00% | 3.60% |
| 20 | Heme biosynthesis (P02746) | 1 | 1.00% | 3.60% |
| 21 | Huntington disease (P00029) | 1 | 1.00% | 3.60% |
| 22 | Heterotrimeric G-protein signaling pathway-Gq alpha and Go alpha mediated pathway (P00027) | 1 | 1.00% | 3.60% |
| 23 | Wnt signaling pathway (P00057) | 2 | 2.00% | 7.10% |
| 24 | Heterotrimeric G-protein signaling pathway-Gi alpha and Gs alpha mediated pathway (P00026) | 1 | 1.00% | 3.60% |
| 25 | 5HT2 type receptor mediated signaling pathway (P04374) | 1 | 1.00% | 3.60% |
| 26 | Hedgehog signaling pathway (P00025) | 1 | 1.00% | 3.60% |
