# Supplementary material for: Proteomic profiling of urinary extracellular vesicles differentiates breast cancer patients from healthy women
Source: PLoS One. 2023 Nov 3;18(11):e0291574. doi: 10.1371/journal.pone.0291574 (PMC10624262; doi:10.1371/journal.pone.0291574)
Supplement: S4 Table — (DOCX) [file pone.0291574.s009.docx]

**S4 Table. UALCAN validation of potential biomarkers from significantly up-regulated uEV proteins in BC compared to CT.**

| **UALCAN** | **DEPs** | | |
| --- | --- | --- | --- |
|  | **Fold change (FC)** | **Log2(FC)** | **Adjusted**  **p-value** |
| 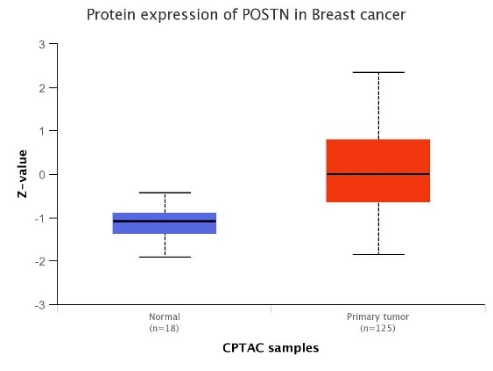  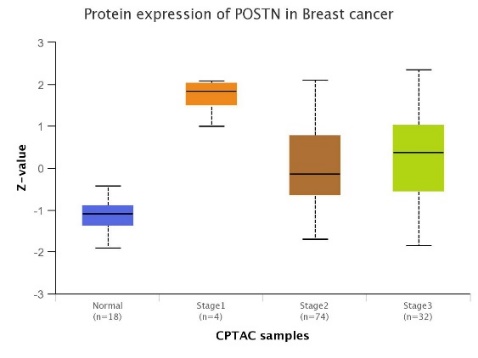 | 20.114 | 4.3301 | 0.02424 |
| 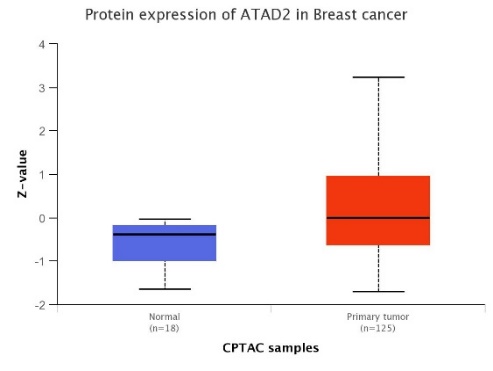 | 20.708 | 4.3721 | 0.00042 |
| 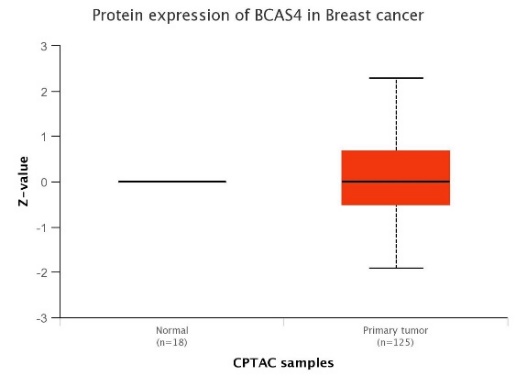 | 6.5886 | 2.72 | 0.004098 |
| 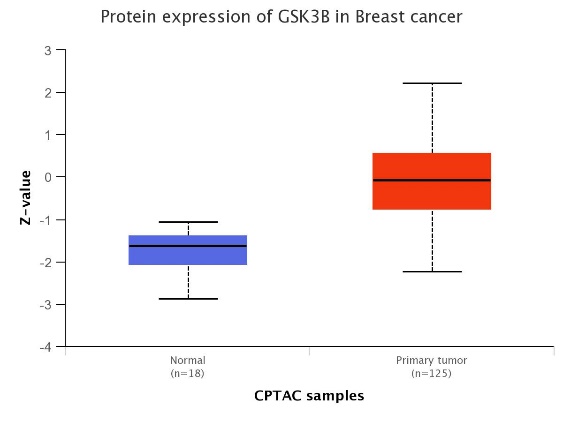 | 12.483 | 3.6419 | 0.00468 |
| 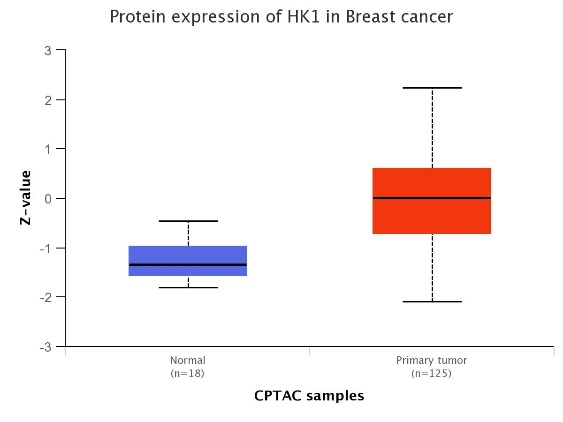 | 9.3514 | 3.2252 | 0.00039 |
| 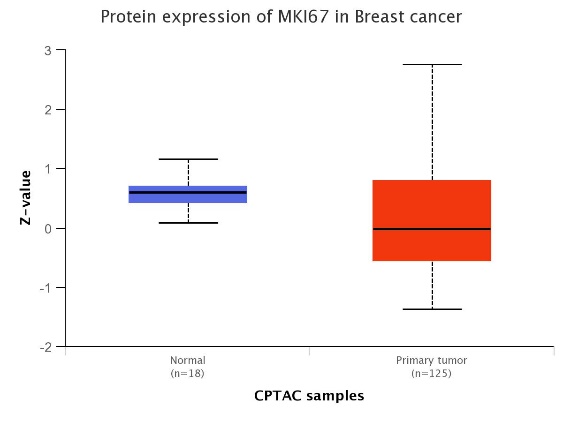 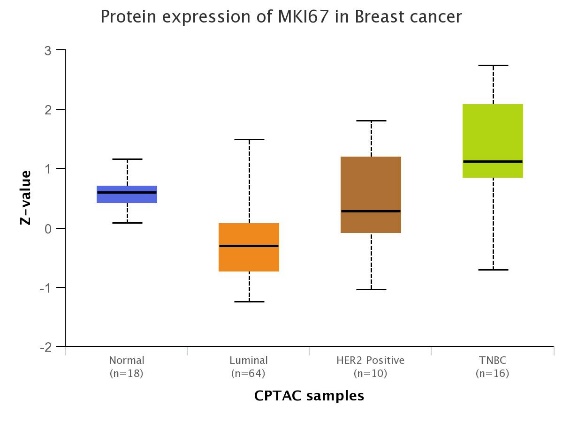 | 32.836 | 5.0372 | 0.002766 |
