# Supplementary material for: Proteomic profiling of urinary extracellular vesicles differentiates breast cancer patients from healthy women
Source: PLoS One. 2023 Nov 3;18(11):e0291574. doi: 10.1371/journal.pone.0291574 (PMC10624262; doi:10.1371/journal.pone.0291574)
Supplement: S5 Table — (DOCX) [file pone.0291574.s010.docx]

**S5 Table. List of unique uEV proteins in different molecular subtypes and stages of BC patients compared to CT.**

| **Control** | **Luminal A** | **Luminal B** | **HER2** | **TNBC** | **Stage I** | **Stage II** | **Stage III** |
| --- | --- | --- | --- | --- | --- | --- | --- |
| Q9H3J6 | Q8NGV0 | Q9NZR4 | P22234 | P05814 | O14717 | P05814 | P39905 |
| P01602 | P39905 | A0A0C4DH33 | P41597 | Q9BVV8 | P22234 | Q8NGV0 | Q63HM9 |
| P62068 | Q8IUB9 | P58004 | P27352 | P61952 | P27352 | Q9Y6C2 | O95639 |
| P78414 | P14151 | Q6MZM9 | Q9Y6C2 | P32247 | Q9NZP0 | Q8IUB9 | Q92572 |
| P0DPK4 | P60409 | Q9H3H9 | Q8N6G2 | O00526 | Q9BVV8 | P60409 | P59190 |
| Q0D2J5 | Q3MHD2 | Q6ZVX9 | Q14165 | O95249 | Q9UPI3 | B6SEH8 | O60762 |
| Q658P3 | Q02083 | Q13889 | C9JN71 | P09693 | P14151 | P28068 | A0A0B4J268 |
| Q8N349 | P50749 | Q8TF64 | Q63HN1 | Q96L08 | Q8N6S5 | Q86VN1 | Q56P42 |
| P20382 | Q86VN1 | Q9BUY5 | B6SEH8 | Q8NGX3 | Q14165 | P14207 | P08571 |
| P35504 | O75414 | Q9NVR7 | P28068 | O95843 | C9JN71 | Q12841 | Q8NGN5 |
| Q9BZL1 | Q63HM9 | P11310 | Q9Y5J7 | Q8IXQ3 | P62888 | Q96KV6 | Q9BRT9 |
| Q2WEN9 | P20916 | Q8IY95 | Q5HYM0 | O75072 | Q63HN1 | Q02747 | P59020 |
| Q9GZY8 | Q96KV6 | Q5JTJ3 | P14207 | O00165 | P08138 | Q8NDZ6 | Q96BW1 |
| P0C866 | Q075Z2 | P63208 | Q96DE0 | P00918 | Q02083 | P61952 | Q96DV4 |
| P22352 | Q5TEU4 | O00425 | Q12841 | P14061 | P50749 | Q5TEU4 | Q9UDV6 |
| Q7Z7H3 | O95639 | Q96BT3 | P16581 | Q86UV7 | O75414 | P32247 | Q9UJG1 |
| P86397 | O75508 | A6H8M9 | P62072 | Q14863 | Q9Y5J7 | O00526 | Q9GZQ3 |
| P60370 | P32881 | Q8TC21 | P17658 | O75908 | Q5HYM0 | P08218 | P25106 |
| P09496 | Q9NQM4 | Q9NRY7 | Q8NCK7 | P10323 | Q96DE0 | P09693 | O15371 |
| Q13884 | P58317 | Q8NCA9 | Q9H207 | Q8WVV9 | P27449 | Q9C0D9 | P58550 |
| Q9UHA2 | P11233 | Q9HB71 | Q9NRJ1 | Q5MNZ9 | P62341 | Q8NH85 | Q86VD9 |
| Q8NGI7 | P14618 | P55212 | P42262 | P56750 | P62072 | O95825 | P60411 |
| Q9Y256 | Q14773 | O00254 | O95825 | Q8WTU0 | A0A0C4DH33 | Q96JK2 | A1L3X0 |
| Q96HY6 | Q9Y2A9 | Q9GZP4 | P20337 | P0CK96 | Q075Z2 | Q7LDG7 | P31260 |
| Q9P272 | Q9BR61 | Q9UMX3 | Q7LDG7 | O14610 | P17658 | O95843 | A6NK58 |
| Q9UHV2 | P41214 | Q86TI4 | O14787 | Q9H9H4 | Q8N428 | Q9HCC9 | P61956 |
| Q58FF8 | P53778 | P56557 | Q9BSH3 | Q16557 | Q9H207 | O75508 | Q5SXH7 |
| Q9UK41 | Q9H0H9 | Q7Z5A9 | Q9NPI6 | Q96DR4 | Q9NRJ1 | P32881 | Q9NWW6 |
| Q9P0P0 | P15036 | Q9NUD9 | Q02505 | Q6ZYL4 | Q96L08 | Q6MZM9 | P05937 |
| Q70SY1 | Q92572 | E5RG02 | O15198 | Q8NDX5 | P20337 | Q8IXQ3 | A0A0D9SF12 |
| Q5VXI9 | O95868 | Q9P0N8 | Q15366 | P59533 | O14787 | Q8N3J9 | P01854 |
| O00590 | Q9BUY7 | A1L157 | Q9NQE9 | Q14916 | Q9HAI6 | Q6FIF0 | Q15744 |
| Q99571 | Q92187 | A6NDU8 | Q17RY0 | O75312 | Q8NGX3 | Q3LI60 | P49789 |
| Q96KX0 | O95168 | A0A096LPI5 | P20248 | Q9NX04 | Q9BZA5 | A6NCC3 | P62952 |
| Q9NTX9 | P52743 | Q9UQ74 | O96008 | Q86SX3 | Q9BSH3 | P11233 | P61236 |
| Q8IXN7 | Q6P988 | Q5SWL8 | Q9H8G2 | Q5SQQ9 | P58004 | P14618 | Q9UPW6 |
| Q8NEU8 | P59190 | Q99712 | A6PVI3 | Q96FV2 | Q9NPI6 | Q14773 | Q9H6Y5 |
| Q14954 | Q5JU69 | X6R8D5 | Q8N5U1 | Q16651 | Q02505 | O75072 | P35244 |
| Q96NX5 | Q8IUX1 | O00321 | Q58G82 | P51575 | O15198 | Q9Y2A9 | Q2NL98 |
| P34925 | Q9BU64 | P50542 | Q8N2I2 | P33260 | Q15366 | O00165 | Q9Y2N7 |
| Q2M3C6 | Q9UH73 | P14770 | Q5QJ74 | E5RJ46 | Q9H3H9 | Q9NQE9 | P61578 |
| Q5JVL4 | O60762 | Q86WR7 | Q401N2 | P19835 | Q8WWB3 | P00918 | P04217 |
| O76003 | Q9Y646 | O95372 | Q6ZRC1 | Q8NCG5 | Q17RY0 | P14061 | P16389 |
| A0A0B4J234 | P00846 | P61576 | Q5J8X5 | P06732 | P20248 | Q86UV7 | O75800 |
| Q8IWA4 | Q9H693 | Q9NWW5 | P11509 | Q96EU7 | Q9BR61 | O96008 | Q96GD0 |
| Q15323 | Q9NU39 | Q2MKA7 | P41182 | Q8IUH5 | Q14863 | Q92819 | Q13304 |
| Q9Y253 | P13861 | Q9BUW7 | P37840 | Q14CZ0 | O75908 | P41214 | Q9NSE2 |
| A6NEK1 | P36543 | A0A075B6J2 | Q9UHA7 | Q496M5 | Q8N5Y8 | Q9H0H9 | Q8WUY1 |
| Q8N8Q3 | A0A075B6I3 | F8WCM5 | Q86T13 | Q6ZP65 | O95868 | A6PVI3 | Q8N8D7 |
| P43308 | Q92581 | P10914 | Q8NGZ3 | Q15819 | P56750 | Q8N5U1 | Q5U649 |
| Q63HM2 | Q9NXU5 | Q8N9W5 | P49901 | O60831 | Q8N2I2 | Q8N9V2 | A0A2R8Y7D0 |
| Q15653 | Q8NEM0 | Q92995 | Q6P9G4 | Q69YU5 | Q401N2 | Q8WVV9 | Q6NTF9 |
| Q8N7N1 | Q9HBZ2 | Q4G0W2 | O76076 | Q96QK8 | Q6ZVX9 | Q5MNZ9 | Q8TD23 |
| Q3LI62 | P0C8F1 | Q17RP2 | A0A1B0GX49 | Q5XLA6 | Q6ZRC1 | Q58G82 | P52739 |
| Q9NVC6 | Q8WTS1 | Q12950 | Q02108 | P42765 | Q8NGK4 | Q5QJ74 | Q9NVR2 |
| Q9UJW9 | Q16082 | Q96HA9 | Q96T88 | A0A0B4J2E0 | Q8N6F8 | Q8WTU0 | P10620 |
| Q01094 | P20226 | P47890 | Q8N8Z8 | Q0VG73 | P41182 | P0CK96 | A9YTQ3 |
| A0A0G2JMI3 | Q6UW88 | Q9H160 | Q8WUE5 | Q6UWP7 | Q86T13 | O14610 | A0A1W2PPF3 |
| Q5VU69 | A0A0B4J268 | O43617 | Q8N976 | Q5TZJ5 | P49901 | Q5J8X5 | P0CB47 |
| Q9H204 | Q5U5Q3 | Q9NXG6 | Q16254 | O95997 | Q6P9G4 | Q13889 | P55058 |
| A6NFZ4 | Q9NS82 | Q92535 | Q9H813 | Q8NGI3 | P0DMT0 | O60725 | Q8TA86 |
| Q9NXV2 | O43914 | Q68DY1 | Q9BYG5 | P39210 | Q6P988 | Q92187 | Q15637 |
| P50583 | Q56P42 | Q9P260 | P13762 | Q99944 | Q02108 | P37840 | O60346 |
| Q9H334 | Q9UBP8 | P25874 | P45877 | Q969V6 | Q8NDX5 | Q9UNN8 | Q7LDI9 |
| P35325 | Q6P2I3 | Q9UDX5 | Q8NFZ4 | P05000 | Q9H009 | Q04760 | Q5JST6 |
| Q8WVK2 | Q5VYS4 | Q8N1D5 | O75964 | Q9BVM4 | Q75MW2 | Q9H9H4 | P63128 |
| P04280 | Q8IVU9 | Q96F86 | Q8TDG2 | Q6ICL7 | P39687 | Q16557 | Q7Z5L9 |
| Q96A09 | Q9H9P8 | Q8NG41 | O60830 | O95800 | Q8WUE5 | O15391 | Q08722 |
| Q2QL34 | Q6P2P2 | O96013 | Q96DT0 | Q9BWW4 | Q14916 | Q8NGZ3 | O75695 |
| Q5VVB8 | Q9H7D7 | O75940 | Q9HAW9 | O95238 | Q9H813 | Q9H3D4 | P59046 |
| Q12765 | Q9BRA2 | P84085 | Q8TE02 | Q96IK0 | Q04941 | Q96DR4 | Q9GZM5 |
| P78385 | O00299 | Q5SZQ8 | O43734 | O00241 | P13762 | A0A1B0GX49 | Q6UXY1 |
| Q8IVG9 | Q96BS2 | Q969L4 | O14994 | Q9BXK1 | O75312 | Q8N8Z8 | Q9Y2C2 |
| Q9NTN3 | Q14106 | Q9NXE8 | O43826 | Q8IV31 | P45877 | Q8IUX1 | Q9Y2T3 |
| Q9NVT9 | Q8TCI5 | Q9H082 | Q9Y547 | P25311 | Q8NFZ4 | P59533 |  |
| P45844 | H3BPF8 | A0A075B6L6 | Q6NUI1 | O75771 | Q16651 | Q9UH73 |  |
| Q5MJ70 | Q8TBM8 | Q9GZM8 | P0CW21 | P32321 | O75964 | Q8N976 |  |
| Q96IR2 | Q8TAG6 | Q9BUI4 | Q9UBF6 | Q96LR4 | Q9NU39 | Q16254 |  |
| P55774 | P08571 | Q8IUR5 | O14656 | Q6SPF0 | P13861 | Q9Y646 |  |
| Q969X1 | Q8NGZ2 | P03956 | Q9NY12 | A0A0B4J279 | P48651 | Q8TF64 |  |
| Q3C1V1 | A0A096LP01 | P15813 | O75896 | Q05952 | P58182 | Q9H693 |  |
| Q86WS3 | P19544 | Q5VT66 | O75388 | P04216 | Q9HAW9 | Q96RQ1 |  |
| Q9H2D1 | Q13131 | Q93091 | P55822 | P63173 | Q8TE02 | Q5SQQ9 |  |
| P52797 | Q8NGN5 | Q14314 | Q8TEE9 | Q6QHF9 | O43734 | Q96FV2 |  |
| P46734 | P62683 | A5PLL7 | A0PK05 | Q12849 | Q96EU7 | P33260 |  |
| Q8WVQ1 | Q9Y6G1 | Q5JU00 | O43488 | Q6UX34 | P0C8F1 | P36543 |  |
| Q9UPY6 | A6NEL3 | P20592 | Q9Y226 | O14880 | Q8IUH5 | Q8TDG2 |  |
| Q8N2C9 | Q7Z4H3 | Q68DY9 | O60383 | A6NJV1 | Q9Y547 | O60830 |  |
| O75333 | Q9BTL3 | Q8N8E1 | O95409 | Q96EA4 | Q8WTS1 | E5RJ46 |  |
| Q5T753 | A6NNH2 | Q99439 | Q9BRP1 | Q01151 | Q9BZ68 | Q9BUY5 |  |
| O60936 | Q3ZCQ2 | Q6P6B1 | Q9H6L2 | Q8N9I0 | P0CW21 | P19835 |  |
| Q8NGS1 | Q8TCH9 | Q08ER8 | O95081 | Q8WVN8 | Q16082 | A0A075B6I3 | |
| Q9UMS0 | Q9Y257 | Q9BVC5 | Q9GZN2 | Q96FQ6 | Q9NY12 | Q92581 |  |
| Q02080 | Q96F10 | Q96EG1 | Q8WUA7 | Q5MNZ6 | O75388 | Q9NXU5 |  |
| Q99878 | Q8NI38 | Q8IV45 | Q9NUM4 | O15389 | P55822 | O14994 |  |
| Q9H6D3 | Q00604 | Q96EK4 | Q13326 | Q9H1N7 | Q8TEE9 | P06732 |  |
| O14772 | Q9BRT9 | Q9Y366 | P55809 | Q8NGI9 | O43914 | Q8NEM0 |  |
| A0A494C0N9 | P21583 | Q86VI1 | P54855 | Q96I82 | A0PK05 | Q9HBZ2 |  |
| A5D6W6 | Q8N695 | A0A1B0GVX0 | Q9Y5H6 | Q13733 | Q9BYN0 | O43826 |  |
| Q9H6E4 | Q9HBU1 | Q9NWM3 | Q6ZT77 | Q6P1R4 | Q9UBP8 | Q14CZ0 |  |
| Q6UWY2 | P59020 | Q9HAJ7 | Q8IUH8 | Q16836 | Q69YU5 | B1APH4 |  |
| Q96PX1 | P0DV79 | A0A1B0GUI7 | Q96IJ6 | P68363 | Q8N6I1 | Q86SJ2 |  |
| P29033 | Q6UW68 | Q8WZA1 | Q6UXK5 | Q8IYI8 | Q6UXS0 | Q496M5 |  |
| Q9Y484 | Q15007 | A6NN92 | Q9Y6Y0 | P09486 | Q5XLA6 | Q6ZP65 |  |
| P06132 | Q86U28 | P40925 | Q6UWF9 | Q9ULR5 | Q99687 | Q6NUI1 |  |
| Q8IV01 | Q9Y6C7 | Q96PN8 | Q8NBV4 | P52738 | O95409 | Q15819 |  |
| Q7L0X0 | Q96BW1 | Q9GZY4 | P0C7T3 | O95257 | A0A0B4J2E0 | O60831 |  |
| A4D0Y5 | Q9Y5N1 | Q8N743 | P63252 | Q8WU39 | Q9H7D7 | Q9UBF6 |  |
| P57052 | P10266 | Q9Y6N1 | P18510 | Q8WWU7 | Q9H6L2 | P20226 |  |
| P04259 | Q9NWS6 | Q9BYG4 | O75888 | P78396 | O00299 | O14656 |  |
| Q9GZV1 | A0A0B4J245 | Q7RTY3 | Q9BVJ7 | Q9H343 | Q6UWP7 | O75896 |  |
| Q6P9F5 | P25089 | P62244 | Q86U10 | Q6DKJ4 | Q9GZN2 | Q5U5Q3 |  |
| Q96T59 | P01732 | Q6P1R3 | Q6UUV9 | A6NFE3 | Q8WUA7 | Q9NS82 |  |
| A6NGZ8 | A0A0C4DH30 | Q96EW2 | Q9NWV4 | Q5W5W9 | Q9GZN6 | O43488 |  |
| P04899 | P52758 | Q6ZUL3 | K7EIQ3 | Q8N1S5 | Q9NUM4 | Q96QK8 |  |
| Q9NSA1 | Q9BZM2 | Q00005 | Q9NVV4 | O43759 | H3BPF8 | O60739 |  |
| Q9BSJ6 | H3BTG2 | Q86WV6 | Q96NU0 | Q8N7C0 | Q5TZJ5 | Q9Y226 |  |
| P14866 | P49771 | P26038 | Q9UM44 | P53004 | Q8TBM8 | Q8IVU9 |  |
| Q9Y4Z2 | Q1EHB4 | P52757 | Q8N1L4 | Q96GX8 | P55809 | Q7Z4L9 |  |
| P01111 | Q5TGI0 | P55008 | O75607 | Q12926 | Q14494 | O60383 |  |
| O60883 | Q96DV4 | P49757 | P30039 | P08754 | P39210 | P42765 |  |
| P31431 | Q9UJH8 | A6NL26 | P61570 | Q8TAD4 | Q8IY95 | Q9BRP1 |  |
| Q5VU36 | P46783 | P32754 | Q5QGZ9 | Q9P0K9 | Q8N138 | Q9NVR7 |  |
| Q86XN8 | Q99541 | Q8TDR2 | Q96LA6 | Q8IWL2 | O95273 | Q9BRA2 |  |
| Q96SM3 | Q99797 | Q86V24 | Q9Y264 | Q8N1E2 | P05000 | Q0VG73 |  |
| Q9NTX7 | P55211 | P00441 | P16662 | Q6ZNC8 | A0A096LP01 | Q6JEL2 |  |
| Q8NGJ8 | Q7Z4W2 | Q8NGS6 | Q9BS18 | Q6PEW0 | Q9Y5H6 | O95081 |  |
| Q9NZH5 | Q9UDV6 | O94900 | A4QPB2 | Q6UWV7 | Q6ICL7 | Q8NEX9 |  |
| Q5JUK2 | O15315 | P43361 | Q96PU9 | Q9UKJ1 | O95800 | Q96BS2 |  |
| O15482 | Q9Y5E7 | Q9BPU9 | Q309B1 | Q9BZW4 | P62683 | Q14106 |  |
| Q96LB0 | Q05923 | P17483 | P51164 | Q06265 | Q96IK0 | Q13326 |  |
| P0CG43 | O75461 | Q9UNM6 | Q07869 | Q5VZ72 | A6NEL3 | Q9BTM9 |  |
| Q8N0Z9 | Q8NC69 | Q9NX40 | Q8NGV6 | O14773 | Q6ZT77 | Q12988 |  |
| Q8NGX2 | P31994 | Q96M63 | P78395 | P20336 | Q8TCH9 | Q86XW9 |  |
| P34130 | P11049 | O43699 | Q96BI1 | P01160 | P25311 | P11310 |  |
| Q8IZD6 | Q9UJG1 | Q9BT56 | Q9UI25 | P15086 | Q6UWF9 | Q99944 |  |
| O00631 | P17947 | Q96C28 | Q6ZSR9 | Q96EZ4 | Q8NBV4 | Q969V6 |  |
| A6NIH7 | Q7L5Y9 | Q9UKW6 | Q3Y452 | A0A0C4DH32 | P0C7T3 | Q5JTJ3 |  |
| Q9P2X8 | Q7Z6M1 | P13805 | Q5RIA9 | Q9BT43 | P32321 | Q8NGZ2 |  |
| Q8IYS1 | Q9P242 | Q6ZMS7 | Q3LI59 | Q9UIV1 | P63252 | P54855 |  |
| Q00403 | Q01628 | Q5BJD5 | P61925 | Q96S96 | P18510 | A0A0B4J266 | |
| A2RU54 | P50221 | Q8NH80 | P58012 | P0CG08 | P21583 | Q9BVM4 |  |
| Q9NS25 | O95007 | Q5W0Q7 | Q9NYX4 | Q9BUA6 | Q9HBU1 | Q8IZL8 |  |
| Q96R48 | C9JQL5 | Q9BUV0 | Q8NGB8 | Q92783 | Q05952 | P02724 |  |
| Q14508 | Q53HC5 | Q14213 | Q3SXZ7 | A8MZ36 | O75888 | Q6ZS02 |  |
| P0DP75 | Q07820 | A0A1W2PPL8 | P0C626 | Q6UXU6 | Q9BVJ7 | Q13131 |  |
| Q8WW62 | Q9Y691 | P48426 | Q9BYQ2 | Q8N9X5 | Q9NUQ2 | Q9BWW4 | |
| Q2WGJ8 | A7E2F4 | O95810 | Q6ZSV7 | Q9BXI9 | Q86U10 | O95238 |  |
| Q9BPW9 | Q9GZQ3 | Q86UU0 | Q9H344 | Q96KC2 | K7EIQ3 | Q9Y6G1 |  |
| Q9NRW3 | P35225 | Q96PC3 | Q9NPR9 | O14817 | P63173 | Q9BTL3 |  |
| Q06055 | O95704 | P55042 | P54922 | Q8NGV5 | Q9NVV4 | Q969D9 |  |
| Q6S5L8 | Q96KP4 | O43869 | Q07507 | A8MWK0 | Q6UW68 | Q96IJ6 |  |
| Q9UBF9 | Q92874 | Q53GA4 | Q9HAW8 | Q9NRW1 | Q8N1L4 | O00241 |  |
| Q9H0W8 | P25106 | Q14397 | Q9NZL4 | O95716 | Q96SI1 | Q3ZCQ2 |  |
| Q8N4S9 | Q8N2M4 |  | Q13072 | Q9BX59 | O14880 | Q9BXK1 |  |
| Q8N5Z5 | P10767 |  | P11245 | Q8NGP0 | A6NJV1 | Q6UXK5 |  |
| P12429 | Q9NYA1 |  | Q9H2H8 | P45983 | Q9Y6C7 | Q8IV31 |  |
| Q7Z3S9 | P50616 |  | Q96GE9 | P59539 | O75607 | Q9Y6Y0 |  |
| Q9HAT8 | Q9H8Q6 |  | Q8NCL8 | Q8N1A6 | Q6J4K2 | O75771 |  |
| O14668 | Q9Y244 |  | P41586 | P24539 | A0A0B4J245 | Q9Y257 |  |
| P08865 | Q6PII3 |  | Q9H6V9 | B2RXH8 | P27037 | Q8NI38 |  |
| Q9HA47 | O15371 |  | P46926 | Q6X9E4 | A0A0C4DH30 | P08174 |  |
| Q96GK7 | Q9NV92 |  | L0R6Q1 | Q16378 | Q8WVN8 | Q00604 |  |
| P10114 | Q9Y6Q6 |  | Q8NGG0 | Q9UL25 | Q96FQ6 | O76000 |  |
| Q9UL33 | P27469 |  | Q9UBF2 | Q8NH16 | Q96LA6 | Q969G6 |  |
| Q8N4W6 | Q13323 |  | Q9BYV2 | Q8N812 | H3BTG2 | Q6SPF0 |  |
| Q8NGF6 | Q9NWZ8 |  | Q96MB7 | Q6NUK4 | Q9BS18 | P63208 |  |
| Q86XT9 | Q99942 |  | P05386 | Q6PEY1 | A4QPB2 | A0A0B4J279 | |
| Q9UN30 | Q2M3M2 |  | Q9ULX9 | O94925 | Q309B1 | Q9BZJ6 |  |
| P98177 | Q16790 |  | Q13166 | Q32M92 | P46783 | Q8N695 |  |
| P22466 | A0A075B6X5 | | P59780 | Q7RTP0 | P51164 | Q9P286 |  |
| Q6ZSY5 | P58550 |  | Q8IUE6 | Q96LB2 | O15315 | Q6UUV9 |  |
| Q86TS9 | Q8N3U1 |  | Q5W064 | Q96EH5 | Q7Z3T8 | Q9NWV4 |  |
| Q96J77 | P01594 |  | Q9NQ92 | P09972 | P78395 | P04216 |  |
| Q9H6W3 | A8MWE9 |  | Q496H8 | Q9C0K1 | P31994 | Q9Y2G5 |  |
| Q16548 | P0DP01 |  | P55055 | Q8WVD5 | Q7L5Y9 | Q6QHF9 |  |
| Q8TBF5 | P53794 |  | Q5SZI1 | Q8NGR2 | Q96BI1 | Q12849 |  |
| Q9NY64 | Q15560 |  | P0DOX3 | A8MX19 | A6NCF5 | Q96NU0 |  |
| P0DME0 | Q86VD9 |  | Q8TCZ2 | Q8NGP9 | Q9P242 | Q9UM44 |  |
| Q5XG99 | P30273 |  | Q17R31 | P01619 | Q16836 | Q3LIE5 |  |
| Q96NS1 | O95498 |  | Q63ZY6 | Q8N1N2 | Q01628 | Q96EA4 |  |
| P15538 | P53611 |  | Q8NCC5 | C9J202 | A6H8M9 | Q9Y5N1 |  |
| Q92947 | Q2TBC4 |  | Q496F6 | Q9GZN4 | Q8TAV3 | P30039 |  |
| O43365 | Q6UWI2 |  | P24522 | Q9Y2D0 | O95007 | P61570 |  |
| Q9UBB6 | Q86TJ5 |  | P54105 | P29459 | Q8IYI8 | Q9NWS6 |  |
| Q8N1Q8 | P50052 |  | Q8TF63 | Q01105 | P61925 | Q96LP6 |  |
| P02776 | Q6UXG3 |  | Q4VCS5 | Q9NR12 | O95257 | Q8N4A0 |  |
| O60635 | Q9NQ40 |  | Q5TG53 | Q9NYR8 | Q9NYX4 | Q01151 |  |
| Q969W8 | P60411 |  | O75841 | Q8TB33 | Q8WWU7 | P25089 |  |
| Q86XJ0 | Q9Y676 |  | P30874 | Q9BQD7 | Q8TC21 | P01732 |  |
| Q6A555 | Q8NHP7 |  | Q9NQY0 | Q9UQB9 | Q9Y691 | Q8N9I0 |  |
| Q8N7U7 | A1L3X0 |  | A6NC42 | Q96FT7 | Q8NGB8 | O43819 |  |
| Q8NG11 | Q9UHY8 |  | Q08116 | C0HLS1 | P0C626 | Q9Y264 |  |
| P03999 | Q92813 |  | Q9BRT8 | Q12829 | A7E2F4 | Q02978 |  |
| P0C862 | Q5STR5 |  | Q9H2R5 | P23760 | Q9NRY7 | P16662 |  |
| Q53QV2 | Q8IUW5 |  | Q9UHL3 | Q6NT16 | Q9BYQ2 | Q9BZM2 |  |
| P56277 | Q8IYP2 |  | Q3LI54 | Q8WV37 | Q6ZSV7 | P49771 |  |
| A4D1S5 | O60294 |  | Q9BT67 | Q14847 | Q92874 | Q5MNZ6 |  |
| O00574 | P21291 |  | Q96E14 | O43903 | Q8N1S5 | Q96PU9 |  |
| Q8NGK3 | P00395 |  | P50553 | Q96PU8 | O43759 | O15389 |  |
| A6NFE2 | Q9UIG4 |  | A8MTJ3 | Q7RTZ2 | Q9NYA1 | O00425 |  |
| Q9C0C6 | Q9HCP6 |  | P19622 | Q9BZM3 | Q8N7C0 | Q5TGI0 |  |
| Q8NGY1 | P81277 |  | Q8NH40 | A4FU28 | Q9Y244 | Q99541 |  |
| Q8NHJ6 | P31260 |  | A6NKD9 | O75449 | Q6PII3 | Q99797 |  |
| Q9Y291 | Q5I0X7 |  | P58170 | P51460 | P54922 | P55211 |  |
| Q14695 | O15393 |  | P59998 | A0A286YF60 | Q9NV92 | Q9H1N7 |  |
| P03915 | A6NK58 |  | P31946 | Q99062 | P0DL12 | Q7Z4W2 |  |
| O00238 | Q6S5H5 |  | Q0PNE2 | Q9BQ13 | Q9HAW8 | Q8NGI9 |  |
| Q1MX18 | A0A286YFK9 | | Q96MM3 | Q8N2X6 | Q9NZL4 | Q05923 |  |
| P22612 | Q8NGA1 |  | Q9Y680 | Q99729 | Q13072 | Q8NGV6 |  |
| Q9NQG1 | Q8TBM7 |  | Q8NGL4 | P35611 | P11245 | Q8NC69 |  |
| P0CG13 | Q8WXH6 |  | P22087 | P36980 | Q96GE9 | Q8NH76 |  |
| Q9NPF4 | P61956 |  | Q9BSM1 | Q9Y6D0 | A0A075B6X5 | Q96I82 |  |
| O75715 | Q13021 |  | Q8WVM7 | P35219 | P41586 | Q7Z6M1 |  |
| P13688 | Q9H094 |  | Q9UHD9 | Q00796 | P46926 | Q9UI25 |  |
| E9PQ53 | Q8WWX0 | | O43296 | Q92956 | Q6PEW0 | Q96BT3 |  |
| P51504 | Q92871 |  | P50148 | P62195 | P30273 | Q6P1R4 |  |
| P40938 | P06454 |  | Q9Y2B1 | H3BQB6 | Q8NGG0 | Q6ZSR9 |  |
| O95452 | P55344 |  | A0A1B0GTI1 | Q6P435 | Q9BYV2 | Q9UKT5 |  |
| P30050 | P58417 |  | Q9NSA3 | Q96MU5 | Q96MB7 | P68363 |  |
| Q8NH56 | Q6UWT4 |  | Q5TF39 | Q96S19 | P05386 | Q3Y452 |  |
| Q5T0B9 | Q8ND25 |  | O00501 | P55087 | Q9ULX9 | Q5RIA9 |  |
| P57059 | Q5SXH7 |  | Q9NRX4 | Q9NX45 | Q2TBC4 | C9JQL5 |  |
| P02689 | Q8NGF3 |  | Q92623 | Q0D2K2 | Q6UWI2 | P09486 |  |
| Q9H477 | A8MV65 |  | A6NNA5 | Q6XLA1 | P19784 | Q9ULR5 |  |
| Q9NX14 | Q9NWW6 | | Q9UGH3 | P10720 | P59780 | P52738 |  |
| Q695T7 | P05937 |  | P0C7L1 | Q9HD33 | Q8IUE6 | P58012 |  |
| Q06546 | Q53HV7 |  | Q8NG97 | O14543 | O14773 | Q8WU39 |  |
| Q14914 | O75792 |  | Q53LP3 | Q9H4I3 | O00254 | P78396 |  |
| Q7RTN6 | Q9H9V4 |  | Q4G148 | Q9BT22 | Q5W064 | Q07820 |  |
| Q9BRV8 | Q6IEV9 |  | Q9NY43 | Q9UI14 | P50052 | P10451 |  |
| P48163 | A0A0D9SF12 | | O95433 | O14757 | P15086 | Q9H343 |  |
| Q8N6D5 | P01854 |  | Q5VZ03 | Q9BWM7 | Q96EZ4 | Q6DKJ4 |  |
| Q6NXT4 | P46020 |  | Q9BWX5 | A0PJX4 | Q5SZI1 | P35225 |  |
| Q8NGI6 | Q15744 |  | P41217 | P14854 | P17181 | Q9H344 |  |
| O00483 | Q5VYV7 |  | Q5JTZ5 | Q8N370 | P0DOX3 | Q8N895 |  |
| Q96T52 | E9PJ23 |  | P15559 | O75348 | Q17R31 | A6NFE3 |  |
| Q6ZR54 | Q96M85 |  | Q9BRI3 | A0A494BZU4 | Q8NCC5 | Q5W5W9 | |
| A9QM74 | A6NGC4 |  | Q9BYJ0 | P08620 | Q96S96 | Q8N2M4 |  |
| Q8IUX4 | Q9Y5G8 |  | P05023 | P43119 | Q86SJ6 | P10767 |  |
| Q6ZTR7 | Q9H4G1 |  | Q9UKY7 | Q9UGI6 | Q9UHY8 | P50616 |  |
| P62851 | Q9NZA1 |  | P16298 | P58513 | Q496F6 | Q9H8Q6 |  |
| P20933 | P49789 |  | P54750 | Q9BT78 | P24522 | P53004 |  |
| P55145 | Q5BIV9 |  | Q8NGA0 | Q6PCB6 | P54105 | Q07507 |  |
| O43187 | P54108 |  | Q96HZ4 | Q96R54 | Q9BUA6 | P27469 |  |
| Q9H7F4 | O60664 |  | Q6IEZ7 | Q16527 | Q9BXI9 | Q9NWZ8 |  |
| P26641 | Q9H3H1 |  | Q6P5S2 | Q8WVB3 | Q8TF63 | Q99942 |  |
| Q9NY56 | P62952 |  | Q96G25 | Q3SY56 | Q5TG53 | Q96GX8 |  |
| Q9UBF1 | P61236 |  | Q9H0M5 | Q8NCT1 | O75841 | Q9H2H8 |  |
| Q9NW68 | Q96NB1 |  | A0A087WSY6 | Q96RJ3 | P16949 | Q2M3M2 |  |
| Q5JWF8 | Q15041 |  | A0A3B3IU63 | Q7RTU3 | A8MWK0 | Q12926 |  |
| Q8NFZ6 | Q3LI77 |  | Q9BS92 | Q9P0V9 | P30874 | P48029 |  |
| Q9BXA5 | Q8IVB4 |  | P67775 | P13349 | O95716 | Q8N3U1 |  |
| Q9NZI7 | Q9UPW6 |  | Q9UKK6 | Q8N1V2 | Q9HCP6 | P01594 |  |
| O95199 | Q9H2J7 |  | Q8TDY3 | Q0Z7S8 | Q7Z5A9 | A8MWE9 |  |
| Q8N0V5 | Q9NRQ5 |  | Q96IM9 | Q9NWS0 | E5RG02 | P08754 |  |
| Q12857 | Q5XKR4 |  | Q9NR50 | O60548 | O15393 | Q8TAD4 |  |
| Q9Y6M9 | Q9Y5F9 |  | Q96HF1 | Q9Y605 | A6NC42 | Q7Z3K6 |  |
| Q03426 | Q9H293 |  | Q96PP4 | O15266 | Q6S5H5 | Q9P0K9 |  |
| Q6A163 | Q6UVW9 |  | Q96EP9 | P21695 | A0A286YFK9 | Q8IWL2 |  |
| Q8WV92 | Q9H6Y5 |  | Q4G0X4 | Q5VWP2 | Q8NGP0 | O14798 |  |
| Q6RUI8 | Q9P2W9 |  | Q96A99 | Q8N8J7 | Q9BRT8 | Q8N1E2 |  |
| P49758 | P35244 |  | Q9UHW9 | P00797 | O60493 | Q15560 |  |
| P78380 | Q9UQB8 |  | P34949 | O15245 | P59539 | Q6ZNC8 |  |
| O15182 | P0DN80 |  | P07738 | P34096 | Q9NQB0 | L0R6Q1 |  |
| P59942 | Q9H1R2 |  | P37235 | Q8IYD1 | Q8N1A6 | Q9HB71 |  |
| Q9Y617 | Q15121 |  | Q4LDR2 | Q5W150 | P06454 | Q6UWV7 |  |
| A0A0C4DH25 | P36897 |  | O75648 | P28324 | O60925 | Q9UKJ1 |  |
| Q16520 | Q9BQS6 |  | Q15388 | Q6ZS62 | Q9UHL3 | Q9UBF2 |  |
| Q6ZUT3 | Q6ICC9 |  | P31274 | Q9BZQ4 | P07315 | O95498 |  |
| P0C7N5 | Q13126 |  | Q05707 | P0DP72 | Q6UWT4 | Q13166 |  |
| Q15506 | Q7L8W6 |  | Q6ZRN7 | Q96Q06 | Q8ND25 | P61916 |  |
| Q96A58 | Q6UXN8 |  | O15540 | Q9H900 | Q9BT67 | Q06265 |  |
| P52895 | Q9UKG4 |  | Q6ZVM7 | P21917 | Q9UL25 | Q5VZ72 |  |
| Q92552 | P42331 |  | P28482 | Q9HD36 | Q6NUK4 | P20336 |  |
| Q6P1N9 | Q6UWD8 |  | P0DPH9 | P19971 | Q7RTP0 | A1A519 |  |
| Q96GV9 | Q1A5X7 |  | O00479 | Q9H1M0 | Q5TBK1 | Q9NQ92 |  |
| O43908 | Q96LS8 |  | Q99578 | A6NC62 | Q9BY41 | Q496H8 |  |
| Q96DW6 | Q6ZQW0 |  | Q8NHW3 | P30968 | P50553 | P55055 |  |
| Q9H9K5 | Q5HYI8 |  | Q8NG84 | Q8WZ59 | A6NDU8 | A0A0C4DH32 | |
| Q7Z6K4 | Q96JA4 |  | O00214 | Q496A3 | A8MTJ3 | Q9GZP4 |  |
| Q8N5I3 | P61366 |  | Q56VL3 | Q8TD94 | Q53HV7 | Q9UMX3 |  |
| O95178 | Q9Y3D0 |  | Q7Z7G2 | Q8N5C1 | O75792 | Q6UXG3 |  |
| P12694 | Q9NWT1 |  | Q03014 | Q71UM5 | P19622 | Q9BT43 |  |
| P05019 | Q9NV35 |  | Q9NUM3 | Q9UNH7 | Q8NH40 | Q8TCZ2 |  |
| A0A1B0GX95 | Q9Y215 |  | Q9BXL5 | Q8NF67 | A6NKD9 | Q9NQ40 |  |
| Q8NGP2 | P17861 |  | O43736 | P15863 | P58170 | Q9UIV1 |  |
| P37088 | Q6ZTN6 |  | P60985 | Q99965 | P59998 | Q9Y676 |  |
| Q8TAI7 | Q3ZCT1 |  | O43749 | Q9UBV8 | A8MX19 | Q8NHP7 |  |
| P55103 | Q3SX64 |  | P51654 | O95456 | A6NJ88 | Q86TI4 |  |
| P06126 | O95644 |  | Q8TAT2 | P32243 | Q8NGP9 | Q9HD64 |  |
| O75531 | Q9UKI8 |  | Q8N4F7 | P60413 | Q9BSM1 | Q92813 |  |
| A8MQB3 | Q9NPI9 |  | Q92551 | Q8N1Q1 | P31645 | Q8IUW5 |  |
| Q8TDV5 | Q9H3P7 |  | Q05329 | Q92485 | Q8WVM7 | Q8IYP2 |  |
| Q99706 | P62699 |  | Q9NZK7 | Q8N9P6 | O60664 | P21291 |  |
| A8MYZ5 | Q9UI42 |  | Q8NHX4 | Q9Y6V7 | C9J202 | P56557 |  |
| Q8IVY1 | O43324 |  | P55056 | Q8N8X9 | Q16799 | P0CG08 |  |
| P31267 | Q8WWV6 | | O14931 | Q8IWE4 | Q9UHD9 | P00395 |  |
| Q9NXZ2 | B3GLJ2 |  | P80217 | Q96R45 | Q96NB1 | Q92783 |  |
| Q8N2C3 | O43739 |  | O14949 | Q8NC44 | O43296 | A8MZ36 |  |
| P52824 | Q6P5R6 |  | Q96B54 | O95278 | O00321 | Q9UIG4 |  |
| Q8WUR7 | Q7Z6V5 |  | Q96LU5 | Q8N8P6 | P50148 | Q6UXU6 |  |
| Q96T55 | Q9NY57 |  | O14807 | Q9Y5A7 | Q5JX71 | Q8N9X5 |  |
| Q6UXN9 | Q9UBN1 |  | Q8NB15 | O95866 | Q5TF39 | Q96KC2 |  |
| Q5HYK3 | Q6IQ49 |  | Q8NCQ2 | A6NNY8 | O00501 | Q4VCS5 |  |
| O75575 | Q96LA8 |  | A6NI15 | Q99811 | Q9NRX4 | A0A0B4J238 | |
| Q8NGD2 | Q2NL98 |  | Q5QJU3 | Q96JY6 | Q01105 | O14817 |  |
| Q9Y3B4 | Q92754 |  | Q7Z5V6 | O75530 | Q9Y5F9 | Q8NGV5 |  |
| Q9NXR1 | P57739 |  | Q2M2I5 | Q9BUK6 | Q92623 | Q9NRW1 |  |
| O95264 | Q5TFE4 |  | A0A0B4J263 | Q9Y2C3 | Q9NYR8 | P81277 |  |
| Q96C11 | P48595 |  | O75380 | Q9Y2K2 | A6NNA5 | P58511 |  |
| Q86SE8 | Q9H0R5 |  | Q9P1Z2 | Q01718 | Q9UGH3 | Q5I0X7 |  |
| B2RXH4 | Q9P0L9 |  | Q969V4 | O95479 | P0C7L1 | Q5SWW7 | |
| Q16581 | Q5W041 |  | Q8ND83 | Q96IG2 | Q9BQD7 | Q9NUD9 |  |
| Q8NGL2 | Q9Y3B1 |  | Q9NV31 | Q9UBR2 | Q9UQB8 | Q9BX59 |  |
| P0DPA3 | P0C629 |  | Q9ULB4 | P32519 | Q8NG97 | Q9NQY0 |  |
| Q8TC12 | Q12947 |  | Q9BYR6 | P20393 | C0HLS1 | Q9NP85 |  |
| Q9NYF3 | Q8NCL9 |  | Q86WC6 | Q9HAP6 | Q53LP3 | Q08116 |  |
| P06239 | Q96NR8 |  | P35625 | Q13158 | P23760 | Q8NGA1 |  |
| P09417 | Q4G0G2 |  | Q9BRC7 | Q9H3S4 | Q9UNP4 | P45983 |  |
| Q6ZN92 | Q9Y2N7 |  | Q9H1Y0 | P14784 | P02655 | Q8TBM7 |  |
| P39877 | E5RIL1 |  | Q9UNZ2 | Q96HY7 | Q5VZ03 | Q9H2R5 |  |
| Q07444 | Q96H78 |  | Q6UWV6 | Q13153 | Q8NGG3 | Q8WXH6 |  |
| Q96BD6 | P47985 |  | Q8NE00 | Q13409 | Q92905 | P24539 |  |
| Q7L0R7 | P61578 |  | Q99895 | P20309 | Q9BZM3 | Q9H094 |  |
| Q717R9 | P29992 |  | Q9Y5G6 | P35070 | Q13126 | Q8WWX0 | |
| P14406 | Q3KP31 |  | P52961 | O15116 | Q9BWX5 | P58417 |  |
| P37268 | P78560 |  | A6NJT0 | Q96RU8 | Q13114 | Q6X9E4 |  |
| Q96CM8 | Q969G2 |  | Q00056 | Q86WC4 | P41217 | Q3LI54 |  |
| Q9BRP4 | Q5BLP8 |  | Q9BXI2 | O43929 | Q5JTZ5 | Q8N2A8 |  |
| Q9Y261 | Q86SX6 |  | Q5XKE5 | Q9NRD1 | Q8N2X6 | Q16378 |  |
| Q8NHH9 | P35241 |  | Q6IPW1 | P10828 | P42331 | Q9P0N8 |  |
| Q6P587 | P45984 |  | P61565 | Q96CS3 | P15559 | Q8NH16 |  |
| P54803 | P51570 |  | P07919 | P24534 | P30046 | Q8N812 |  |
| P08962 | Q9H2W1 |  | Q8NAJ2 | Q5EE01 | Q9BRI3 | Q6PEY1 |  |
| Q8IVU1 | Q9NQ33 |  | Q3SY46 | Q96NL0 | Q1A5X7 | A6NLX3 |  |
| O15069 | P61966 |  | Q9UKI2 | A0A1B0GUS0 | Q96LS8 | O94925 |  |
| P62913 | Q9UBI6 |  | P17213 | Q86U37 | Q9BR26 | Q32M92 |  |
| O95190 | P35318 |  | Q8TBG4 | Q9BTK6 | Q8NGA0 | A1L157 |  |
| Q13492 | O75438 |  | Q9H244 | O00233 | Q00796 | Q96E14 |  |
| Q6GMV3 | Q13948 |  | Q96EX1 | Q5JTC6 | Q96HZ4 | P13725 |  |
| P54868 | P51686 |  | Q9H0C8 | Q8NA42 | Q9H6B1 | Q96LB2 |  |
| O14618 | P31213 |  | Q86XP1 | P15814 | Q96G25 | A8MV65 |  |
| Q3KQV3 | Q9BRK0 |  | Q92945 | Q9NNX1 | A0A087WSY6 | Q96A46 |  |
| O43396 | Q9NPD8 |  | Q8WW32 | Q99616 | A0A3B3IU63 | Q96EH5 |  |
| O96015 | P55851 |  | Q8N8Z3 | Q8IZP2 | Q9BS92 | A0A096LPI5 | |
| Q10981 | O96001 |  | Q3LXA3 | Q96QH2 | P67775 | Q9H9V4 |  |
| Q8NBV8 | Q8TEY5 |  | Q9Y2G1 | Q8IZL2 | Q9UKK6 | P09972 |  |
| Q6XYQ8 | Q96EY8 |  | O75821 | P37288 | P0C617 | Q9UQ74 |  |
| Q14142 | P56282 |  | P13987 | Q9NZJ7 | Q9BT22 | Q9Y5K5 |  |
| Q5TAA0 | Q5XUX0 |  | Q8NE65 | P61201 | Q01860 | Q8NGI2 |  |
| Q9NP59 | Q8NFU0 |  | Q96CK0 | O14753 | Q96IM9 | Q5VYV7 |  |
| Q9NX01 | Q03181 |  | Q8NGT2 | C9J1S8 | Q9NR50 | E9PJ23 |  |
| Q9Y2H1 | Q9UM63 |  | P12525 | Q8N9L7 | Q96HF1 | Q29980 |  |
| Q13258 | Q96CG3 |  | O75712 | Q96DG6 | Q96PP4 | P31946 |  |
| Q8TC44 | Q4G1C9 |  | Q9BZ98 | A0A1B0GVG6 | A0PJX4 | Q99712 |  |
| P29274 | Q8IUC1 |  | P0DM35 | Q15842 | Q96EP9 | Q0PNE2 |  |
| Q9BY77 | Q969Y0 |  | Q12982 | Q96P70 | Q96A99 | Q9C0K1 |  |
| Q15814 | Q00535 |  | P09234 | Q9UKT8 | P08620 | Q96M85 |  |
| Q9NRN9 | Q6UX73 |  | Q96EX3 | Q8TB69 | Q6PCB6 | Q9Y680 |  |
| Q13319 | Q1HG44 |  | Q13561 | Q13332 | P07738 | Q8NGL4 |  |
| Q9Y6W6 | Q16654 |  | Q9UH92 | P62906 | Q16527 | Q9Y5G8 |  |
| Q07699 | Q0P670 |  | Q5T681 | Q3ZAQ7 | P37235 | Q9NZA1 |  |
| Q9NR19 | Q7KYR7 |  | P23582 | Q5VT99 | Q4LDR2 | P01619 |  |
| O60826 | Q9UFV1 |  | Q6ZNA5 | Q5GH76 | O75648 | X6R8D5 |  |
| Q9Y6H3 | A6NEY8 |  | Q96AP7 | Q96EL3 | Q15388 | P38405 |  |
| P39059 | Q9Y6G3 |  | Q7Z7M1 | Q9P127 | Q8WZ55 | P54108 |  |
| Q6P087 | P62987 |  | P10124 | A6NKN8 | Q9P0V9 | Q9H3H1 |  |
| A7XYQ1 | Q6PIF2 |  | A6NL08 | Q9BXW4 | Q5W041 | Q9Y5C1 |  |
| Q2VPK5 | Q9Y3E7 |  | Q15006 | Q5VVY1 | P13349 | Q9GZN4 |  |
| Q8N0V3 | P01566 |  | Q9BYX7 | Q9P2I0 | Q9BZP6 | Q9Y2D0 |  |
| Q9H7T9 | A0A1W2PPG7 | | P13797 | P16871 | Q8N1V2 | Q9H1B7 |  |
| O43670 | P04217 |  | Q9BU89 | P19961 | Q96NR8 | P29459 |  |
| P60321 | Q8N0U4 |  | Q9NUQ3 | Q9Y6F7 | O00479 | Q15041 |  |
| Q9Y5Y2 | Q99798 |  | Q8TE67 | P22626 | Q99578 | Q8IVB4 |  |
| Q6UXB2 | Q9ULR0 |  | P0CF75 | Q13547 | Q8NHW3 | A0A1B0GTI1 | |
| Q8NDX2 | Q8WVF5 |  | Q9H9A5 | Q6ZTW0 | Q8NG84 | Q9NSA3 |  |
| Q9H6F5 | Q9H7B7 |  | Q96KB5 | Q8TAA1 | Q56VL3 | P14770 |  |
| A6NLP5 | Q8N5M1 |  | Q8IYG6 | Q8NFD4 | Q7Z7G2 | Q9NR12 |  |
| O14492 | P09471 |  | Q9HD26 | Q9H1A7 | Q5VV17 | Q86WR7 |  |
| Q6P9F7 | Q6NXE6 |  | Q9Y2S7 | O15554 | Q03014 | Q9H293 |  |
| Q96A08 | Q9UM21 |  | Q99755 | Q96IW2 | O43736 | O95372 |  |
| P57682 | P0DTF9 |  | Q6AI39 | A6NIU2 | P47985 | Q6UVW9 |  |
| Q9UJA9 | P41143 |  | Q12904 | O60641 | Q8N5N7 | Q9P2W9 |  |
| Q9Y548 | Q5VWZ2 |  | Q13795 | O43405 | Q8N8J7 | Q8TB33 |  |
| Q9BVL2 | Q96IW7 |  | Q6ICB4 | Q9P2F6 | A0A075B6H8 | P55957 |  |
| Q86VR7 | O75628 |  | Q9BPZ7 | Q12792 | Q8TAT2 | Q9H1R2 |  |
| O75409 | P48739 |  | Q6IF99 | Q8TAG5 | Q8N4F7 | Q96FT7 |  |
| P48380 | Q8NI29 |  | O60259 | Q8TAV4 | Q969G2 | Q4G148 |  |
| A6NJW4 | Q00G26 |  | Q8TBB5 | Q6RFH5 | Q05329 | Q9NY43 |  |
| Q86TJ2 | Q9Y2D2 |  | Q9NW82 | Q8NHC4 | O15245 | Q12829 |  |
| Q13491 | Q9UC06 |  | Q9Y6S9 | Q8NFI4 | Q96B77 | Q6NT16 |  |
| Q99880 | A0A1B0GVG4 | | Q9NQC3 | P55160 | Q8NHX4 | O95433 |  |
| P55263 | Q96S21 |  | Q15800 | A2RUH7 | P55056 | Q9BQS6 |  |
| Q9NP87 | Q86YL5 |  | Q7L1S5 | P61353 | O14931 | Q8WV37 |  |
| Q13049 | Q9NNW7 |  | Q96KP1 | Q9BQE5 | Q96B54 | Q14847 |  |
| Q8NGT0 | O60359 |  | P60153 | Q96GY0 | Q96LU5 | Q6ICC9 |  |
| P0CH99 | P51858 |  | B2RXF5 | Q96IR7 | Q9BZQ4 | O43903 |  |
| P57086 | Q9NX08 |  | Q9C056 | Q9H0A9 | P51570 | Q7RTZ2 |  |
| Q6P5Q4 | Q8NGK9 |  | P01178 | Q86VD7 | O14807 | A4FU28 |  |
| Q9H4A4 | Q2TAM9 |  | Q5SQ64 | Q9UNA1 | P61966 | P50053 |  |
| Q9HD90 | P16389 |  | Q9NP90 | Q9GZS0 | Q9UBI6 | Q8N7R1 |  |
| Q6PEV8 | O75800 |  | Q6PIS1 | Q8N123 | Q5QJU3 | P61576 |  |
| A8MUI8 | Q8IYV9 |  | Q8TEA1 | Q9Y225 | Q9NPD8 | O75449 |  |
| O95985 | O95707 |  | Q9UIM3 | Q96IY1 | A0A0B4J263 | P51460 |  |
| Q8IZQ8 | Q5QGT7 |  | Q96T21 | Q8NG35 | Q8N5C1 | A0A286YF60 | |
| Q96KK4 | P09661 |  | Q7Z7J9 | Q9P215 | P29275 | Q99062 |  |
| Q9NYQ3 | Q9Y575 |  | Q68EN5 | A2RUC4 | O75380 | Q9UKG4 |  |
| A4D263 | Q99952 |  | Q9UG22 | O76015 | P55851 | Q9BQ13 |  |
| Q5U623 | Q13416 |  | Q92828 | P55039 | Q9P260 | P35611 |  |
| O14786 | Q96GD0 |  | Q6AWC2 | Q8IWZ4 | Q8WWG9 | Q16206 |  |
| Q6KF10 | Q9Y4E1 |  | P69905 | Q8IU54 | Q969V4 | Q8NEP9 |  |
| O60547 | P52597 |  | Q8IVW1 | Q8N8I6 | Q96EY8 | Q9BYJ0 |  |
| Q9H875 | Q6P1Q0 |  | Q5JQF8 | Q68CK6 | P56282 | Q6ZMS4 |  |
| O95876 | Q8TCT8 |  | P36957 | A6NED2 | Q5XUX0 | P05023 |  |
| Q9Y3B8 | P01100 |  | Q6NSI8 | Q2MJR0 | Q8ND83 | O95571 |  |
| A6NFN9 | Q9UGV2 |  | Q02094 | Q14129 | Q9UNH7 | Q9UKY7 |  |
| Q8NGR4 | P53672 |  | Q9UKY4 | Q7L8S5 | Q8NF67 | P16298 |  |
| Q9H1P3 | Q5JRS4 |  | Q9UII5 | P59282 | P15863 | P36980 |  |
| A5PLN7 | Q5MJ68 |  | Q96GE6 | P08311 | Q03181 | Q9Y6D0 |  |
| A2RRD8 | A1L190 |  | Q9GZX7 | Q7Z5U6 | Q9UM63 | P35219 |  |
| P67809 | Q14469 |  | O00220 | Q9NWX6 | Q9ULB4 | P01148 |  |
| A6NF36 | Q9NVH1 |  | Q49A17 | Q9Y271 | Q8IUC1 | Q92956 |  |
| P30626 | P0C7T7 |  | P11498 | Q9UK13 | P32243 | P62195 |  |
| Q96J88 | O15213 |  | Q9NUL3 | P98170 | P35625 | Q2MKA7 |  |
| Q96M27 | Q13304 |  | Q9Y4A0 | Q13564 | Q9UDX5 | Q6IEZ7 |  |
| Q8IVB5 | P49720 |  | Q8TD31 | Q86XF0 | P08185 | Q96JA4 |  |
| Q9H8Y5 | Q8N130 |  | O43543 | A6NKX1 | P60413 | H3BQB6 |  |
| Q8N807 | Q9UQC2 |  | P56975 | P30405 | O75525 | Q6P435 |  |
| Q3SY00 | Q15617 |  | P02647 | Q9H074 | Q8N1D5 | Q96MU5 |  |
| O96024 | P0DV77 |  | Q9Y388 | Q9HAE3 | Q96F86 | Q1T7F1 |  |
| Q96LW4 | O43829 |  | Q8NA77 | Q9UBK8 | Q9H1Y0 | Q9UBR5 |  |
| Q9Y314 | Q9Y337 |  | A0A5B6 | Q9UIJ5 | Q9Y6V7 | Q15040 |  |
| O76095 | Q8N888 |  | Q96A47 | P10909 | A6NEY8 | P55087 |  |
| Q96NM4 | P17844 |  | Q5VWJ9 | P11684 | Q96R45 | Q9BUW7 |  |
| Q9BTY2 | P49748 |  | O76070 | P67870 | Q9UNZ2 | Q6P5S2 |  |
| Q6MZN7 | O60704 |  | Q9C0K0 | Q02575 | Q99895 | A0A075B6J2 | |
| Q8IZJ4 | Q8WXD2 |  | Q9NRC8 | Q96CT7 | Q5SZQ8 | Q9NX45 |  |
| Q9BY76 | Q9NQZ6 |  | C0HM02 | O60299 | Q8N8P6 | Q0D2K2 |  |
| C9JVW0 | Q96SL4 |  | Q92963 | P54845 | Q9Y5G6 | Q9NV35 |  |
| Q06828 | Q9NP95 |  | Q5VVP1 | Q6N063 | Q8N0U4 | F8WCM5 |  |
| Q9NWT6 | Q9NXE4 |  | P20073 | Q9Y5G0 | Q969L4 | Q16204 |  |
| O00481 | Q16891 |  | Q96D71 | Q9Y4K0 | P52961 | Q6XLA1 |  |
| Q9H672 | Q9H015 |  | Q03135 | Q7L8J4 | Q00056 | P10914 |  |
| P49914 | Q155Q3 |  | Q9Y2J8 | A0A075B6I0 | Q9BXI2 | Q99689 |  |
| Q8N6N6 | Q9NSB8 |  | Q1W4C9 | P78330 | Q5XKE5 | Q8NEQ6 |  |
| P99999 | O76013 |  | Q5TA78 | Q9NWA0 | P09471 | P21589 |  |
| Q13232 | Q9NSE2 |  | Q8N9U9 | Q9H0X4 | A0A075B6L6 | P10720 |  |
| Q9NVF7 | Q9NZZ3 |  | Q8WXF3 | A6NHQ4 | Q9UM21 | O14543 |  |
| Q9NRW7 | Q6UXC1 |  | Q9BYG8 | Q8NA92 | Q9UKX7 | O95644 |  |
| Q13895 | A4D2B8 |  | Q8NGP4 | Q9H2S6 | P07919 | Q9H4I3 |  |
| Q96MH7 | Q9UBD0 |  | Q8IYA7 | Q8N3C7 | Q3SY46 | Q9NS86 |  |
| Q8NFY9 | O60688 |  | Q8TEV9 | Q9HAC8 | Q5VWZ2 | Q9NPI9 |  |
| Q2VPJ9 | Q8IVW8 |  | Q8N7X4 | Q8N7R7 | Q9UKI2 | Q9UI14 |  |
| Q9H169 | P13010 |  | Q92537 | P08697 | Q8TBG4 | P62699 |  |
| Q7Z4V0 | Q9H3G5 |  | A6NLE4 | Q5VZ52 | Q8NI29 | Q9UI42 |  |
| Q3KPI0 | Q9Y2A7 |  | Q9UHC3 | Q9UHY7 | Q9H244 | O14757 |  |
| Q96Q83 | P48436 |  | P02794 | Q9HDB5 | Q96EX1 | Q8NGV7 |  |
| O95231 | O00458 |  | P57081 | Q7Z418 | Q9H0C8 | Q8WWV6 | |
| Q8NCD3 | Q8IVS8 |  | Q8WUU4 | A0A1B0GUS4 | Q92945 | B3GLJ2 |  |
| P09543 | Q9Y5F1 |  | Q8NGD3 | Q86UD7 | Q8IUR5 | O43739 |  |
| P15291 | Q6UXU4 |  | Q92765 | Q3SY05 | Q8WW32 | Q92995 |  |
| O15194 | Q8TF21 |  | Q9BXJ3 | Q96BW5 | Q3LXA3 | Q9BWM7 | |
| O15524 | Q9GZK4 |  | P52564 | C9JR72 | Q01718 | O75593 |  |
| Q9UMS4 | Q01813 |  | Q96BK5 | O95674 | Q96IG2 | Q5T0T0 |  |
| Q9Y586 | Q15628 |  | Q8NEP7 | O15260 | Q9H5J8 | O75956 |  |
| Q92968 | Q16877 |  | P08253 | Q63HQ0 | P32519 | Q6P5R6 |  |
| O94993 | Q9Y6K8 |  | Q96MF7 | O95867 | P13987 | P14854 |  |
| P15514 | Q330K2 |  | Q14642 | Q8TBF2 | Q13158 | Q7Z6V5 |  |
| Q13188 | Q9Y2T7 |  | Q6UW49 | A8MXE2 | Q8NE65 | Q8N370 |  |
| P17482 | Q8WUY1 |  | Q96GM8 | Q5U651 | Q5QGT7 | A0A494BZU4 | |
| P06493 | Q6NT76 |  | Q96PY0 | O75820 | O75712 | Q9BZW5 |  |
| Q8N9S9 | P04183 |  | Q86VF5 | Q6UXH8 | P09234 | Q9UBN1 |  |
| P30953 | O76099 |  | Q9H210 | Q60I27 | O60928 | Q6IQ49 |  |
| Q9Y224 | Q8NEW7 |  | Q9UHK0 | Q96PB7 | O15116 | P43119 |  |
| Q9H2F9 | Q9UK85 |  | Q8IYJ2 | Q8N6L0 | Q96RU8 | Q9UHW9 |  |
| O00186 | Q8N8D7 |  | Q5VYX0 | Q8WUF8 | Q9UH92 | Q96LA8 |  |
| Q12959 | Q3KNS1 |  | P57768 | O75462 | Q9NRD1 | Q9UGI6 |  |
| Q9UBX1 | Q66K64 |  | P83859 | O75631 | Q9UGV2 | P58513 |  |
| P43629 | Q6ZWB6 |  | P59095 | Q96DE9 | P08247 | Q4G0W2 |  |
| Q15334 | A6NIJ9 |  | Q15846 | Q9BZL3 | Q5MJ68 | Q8TDV0 |  |
| P41567 | Q14094 |  | Q5TA89 | P01127 | Q52M75 | Q96R54 |  |
| Q99633 | Q8N531 |  | A6NIN4 | Q9BXM9 | P23582 | Q8WVB3 |  |
| Q9BWW8 | Q14894 |  | Q9H0A6 | P60880 | Q6ZNA5 | Q3SY56 |  |
| Q9H3U5 | O75838 |  | Q00444 | Q9Y2T2 | Q5JU00 | A0A3B3IT52 | |
| P61296 | P05543 |  | A0A286YF58 | O75818 | P10124 | Q8NCT1 |  |
| Q13203 | Q05066 |  | Q96C92 | Q9H560 | P49720 | Q5TFE4 |  |
| Q7RTS9 | Q5GAN3 |  | Q8N344 | Q9NX62 | A6NL08 | Q9UNT1 |  |
| Q8TD10 | P20809 |  | A7MD48 | P52306 | Q5EE01 | Q96RJ3 |  |
| Q9H598 | Q5T6J7 |  | O00217 | Q86TL0 | Q96S55 | P31274 |  |
| Q9NP50 | Q5U649 |  | O43174 | Q92481 | Q9UQC2 | P48595 |  |
| Q8IYS0 | Q14681 |  | Q96L46 | O15541 | O00233 | Q7RTU3 |  |
| A6NCV1 | Q96MT8 |  | P15391 | O15392 | Q9BYX7 | Q9H0R5 |  |
| Q9UJT0 | A0A2R8Y7D0 | | Q8NE28 | Q9UFG5 | Q5JTC6 | O15540 |  |
| O95626 | Q92731 |  | Q6ZSA7 | Q96C01 | P13797 | Q8NGG1 |  |
| Q9UJK0 | P60896 |  | Q9Y221 | A6NHZ5 | P15814 | Q9P0L9 |  |
| O15243 | Q8N323 |  | Q8WTT0 | P62857 | Q9NUQ3 | Q9Y3B1 |  |
| Q5T1N1 | O95405 |  | Q15649 | P34896 | Q8WXD2 | Q12947 |  |
| A0A1W2PR82 | Q8WV93 |  | O15321 | P15976 | Q99616 | Q0Z7S8 |  |
| P61024 | Q9Y6E0 |  | Q6ZMT1 | Q16698 | Q8TE67 | Q8NCL9 |  |
| Q86TA1 | Q6NTF9 |  | Q7Z5H4 | P02760 | P23381 | Q6ZVM7 |  |
| Q7L3T8 | P52945 |  | Q9Y5E3 | Q9HCK0 | Q9NP95 | Q9NWS0 |  |
| Q86YS7 | Q5VTH2 |  | Q6UXB8 | Q7RTX9 | P0CF75 | P0DPH9 |  |
| Q9BZM5 | Q8N814 |  | Q8N2Z9 | Q969P0 | P14555 | Q6NXP6 |  |
| Q6ZQR2 | Q8N9V6 |  | P0DJH9 | Q75NE6 | Q8N8E1 | O60548 |  |
| P58294 | A0A1B0GVQ3 | | Q12934 | O75486 | Q9P278 | Q9Y605 |  |
| Q96LJ8 | Q8TAD2 |  | Q9GZY1 | Q69YN2 | Q96KB5 | Q569K6 |  |
| Q9NR16 | Q9Y2L5 |  | Q9Y227 | Q9Y6I7 | Q9H015 | O15266 |  |
| Q9Y2W7 | Q9UDV7 |  | Q6ZNG2 | Q8IX29 | Q9Y2S7 | Q9NUM3 |  |
| Q86TI2 | Q9NPD3 |  | Q9BQP9 | Q8NDQ6 | Q9BWT6 | E5RIL1 |  |
| Q6PK04 | Q9HB89 |  | O95319 | P49419 | Q6P6B1 | P21695 |  |
| Q96M02 | A0A1W2PR19 | | Q8TDN6 | Q9NQ75 | Q6UXC1 | Q5VWP2 |  |
| O76061 | P48741 |  | P17787 | Q96JB8 | Q9UBD0 | P60985 |  |
| P08727 | Q99750 |  | Q9P2G3 | Q8NH60 | Q9NPF5 | Q8N443 |  |
| P20472 | Q6ZMJ4 |  | Q9UIH9 | Q96M66 | Q6AI39 | P40425 |  |
| P21918 | O14967 |  | Q15759 | Q9NS93 | Q9NZJ7 | P51654 |  |
| Q8N4T8 | Q8WWY7 | | P56555 | Q9Y625 | Q8IVW8 | Q96HA9 |  |
| Q8NGN0 | P26885 |  | P01009 | Q9NX36 | Q9BVC5 | Q3KP31 |  |
| P46976 | Q8TD23 |  | Q7Z2E3 | Q96N03 | Q8IVT5 | Q92551 |  |
| Q8NG78 | Q96H20 |  | Q6ZSZ6 | P38646 | P48436 | P00797 |  |
| P10644 | P15248 |  | Q9NP08 | A6NI79 | Q13795 | Q9H160 |  |
| Q8TF39 | E9PB15 |  | Q9NVS2 | P55040 | Q49AG3 | Q5BLP8 |  |
| Q93045 | P52739 |  | P30542 | P50440 | Q9BPZ7 | P34096 |  |
| P83105 | Q86T29 |  | Q6UWH4 | Q96JP9 | O60259 | Q86SX6 |  |
| P04554 | Q8TF76 |  | O75781 | Q12840 | Q8TBB5 | Q8IYD1 |  |
| P36954 | P07093 |  | P34903 | P10073 | Q9NW82 | Q6PIU2 |  |
| Q8N402 | Q9NVR2 |  | Q96HJ9 | Q8WXB4 | Q6UXU4 | Q5W150 |  |
| Q9BW62 | Q9UIG8 |  | P05154 | Q7Z6P3 | Q15628 | P35326 |  |
| Q86UN3 | Q14201 |  | Q8IX05 | P10109 | Q9NQC3 | Q6ZS62 |  |
| Q6RW13 | Q6IN84 |  | P36542 | Q8IXF9 | Q7Z3Z4 | P35241 |  |
| Q5XKP0 | Q9BR76 |  | Q96NG8 | Q8NA75 | Q9UKT8 | P45984 |  |
| P18825 | P0DKX0 |  | Q9Y2I6 | A6NC05 | Q15800 | P0DP72 |  |
| Q15722 | Q8NEA4 |  | Q96K30 | Q92570 | Q8TB69 | Q96Q06 |  |
| A6NJZ3 | P10620 |  | P43351 | Q9ULR3 | Q9Y366 | Q9NW81 |  |
| Q06210 | P35354 |  | Q6AZW8 | P62937 | P82094 | O43617 |  |
| Q9H209 | Q8IU85 |  | P20273 | P29972 | Q330K2 | P21917 |  |
| Q96ND0 | Q9H336 |  | Q6F5E7 | Q9GZP7 | P80162 | Q9HD36 |  |
| Q96AX2 | A1A4Y4 |  | P43365 | Q5TFG8 | P60153 | P19971 |  |
| Q86WK7 | Q9H8H3 |  | Q9BXJ2 | Q9H773 | B2RXF5 | Q8NB15 |  |
| Q9BZE2 | Q5VZF2 |  | Q9UHA3 | Q6UN15 | Q9C056 | Q6E213 |  |
| P08842 | P34931 |  | Q6UW01 | O60894 | P01178 | Q8NCQ2 |  |
| Q9Y2G7 | Q9Y287 |  | Q9NQ32 | Q9P299 | A6NKN8 | Q92535 |  |
| Q8N9I5 | A9YTQ3 |  | Q96KN8 | Q9NR21 | Q5SQ64 | Q9H1M0 |  |
| Q9H2S9 | Q9BVC3 |  | Q9H158 | Q8N9L1 | P01213 | A6NC62 |  |
| Q96A73 | Q8N7B1 |  | P28562 | A6NJI1 | P04183 | P30968 |  |
| Q16772 | P17041 |  | Q86X60 | P13473 | P16871 | O75438 |  |
| Q9BQQ7 | Q9Y4X0 |  | A0A1B0GUX0 | P18283 | Q8TEA1 | Q8WZ59 |  |
| P52954 | P82932 |  | P08246 | Q9HCL3 | Q9UIM3 | Q13948 |  |
| Q9BUA3 | Q86WN1 |  | Q99986 | Q8IYT1 | Q86UA1 | A6NI15 |  |
| Q969Q0 | Q8NCK3 |  | Q6PD74 | Q9HCL0 | Q7Z7J9 | P51686 |  |
| Q6UXI7 | Q8WU17 |  | Q9BXW7 | Q629K1 | Q9Y6F7 | Q496A3 |  |
| Q6ZN18 | Q14353 |  | Q13309 | Q9BSN7 | P22626 | Q8TD94 |  |
| Q96IQ7 | Q9P1U0 |  | Q13303 | P27815 | Q68EN5 | Q7Z5V6 |  |
| P21673 | Q5VT33 |  | P38606 | Q9HCY8 | Q6ZWB6 | Q68DY1 |  |
| A0A075B6Y3 | Q9H106 |  | P11831 | Q7Z4H4 | Q9UG22 | Q14320 |  |
| P69208 | P19525 |  | Q05048 | P36222 | Q92828 | O96001 |  |
| O14828 | Q5SV17 |  | A8K7I4 | Q9HBL6 | Q6AWC2 | Q8TEY5 |  |
| Q8IXQ9 | A2RU30 |  | P29034 | Q9H6J7 | P69905 | Q71UM5 |  |
|  | Q13515 |  | Q9UNX3 | Q13352 | Q8IVW1 | Q9Y6F6 |  |
|  | P30304 |  | Q9H3Y6 | Q14118 | Q5JQF8 | Q0D2I5 |  |
|  | A8MWD9 |  | O76075 | P04629 | P36957 | Q9NV31 |  |
|  | Q8N6Q8 |  | P43304 | P48051 | Q68DH5 | P25874 |  |
|  | Q8IZ40 |  | O75521 | Q13938 | Q6NSI8 | Q9BV97 |  |
|  | Q96J92 |  | Q5T124 | Q5T089 | Q9UII5 | Q99965 |  |
|  | Q32NC0 |  | Q8WZ71 | O43854 | Q96GE6 | Q9UBV8 |  |
|  | Q6PJQ5 |  | O43399 | Q15014 | Q05066 | Q5TDP6 |  |
|  | Q86Z02 |  | P14317 | Q53GS7 | O00220 | Q4G1C9 |  |
|  | A6NGU7 |  | Q86UD4 | Q9H7B2 | Q49A17 | Q14623 |  |
|  | O95863 |  | O75884 | P22304 | O43405 | Q00535 |  |
|  | Q9H8S9 |  | Q92917 | Q9Y240 | Q9NUL3 | Q86WC6 |  |
|  | Q9NYL2 |  | Q9BQB6 | O75674 | Q8WZA1 | O95456 |  |
|  | A0A1W2PPF3 | | A1L167 | Q8WUD4 | O43543 | Q8WYQ4 |  |
|  | P35227 |  | Q9UK08 | Q8N183 | O14771 | Q1HG44 |  |
|  | Q13495 |  | Q5D0E6 | P37287 | P56975 | Q9NUB4 |  |
|  | Q8IUQ4 |  | P35557 | A5YM72 | P02647 | Q16654 |  |
|  | Q9P0J1 |  | Q13227 | Q8WUJ1 | Q9P2F6 | Q8N1Q1 |  |
|  | P11940 |  | Q9NP72 | Q9NRP4 | Q8TAG5 | Q9BRC7 |  |
|  | Q6ZRR5 |  | Q03938 | Q6UWN5 | Q8NA77 | Q8N801 |  |
|  | Q9BQ16 |  | Q09028 | Q9Y3M2 | A0A5B6 | Q92485 |  |
|  | P0CB47 |  | Q86W34 | A4D256 | Q96A47 | P0CW20 |  |
|  | P55058 |  | Q9NZ72 | Q8NGS0 | Q8N323 | Q8N9P6 |  |
|  | Q9Y397 |  | Q6PI78 | P31995 | Q7Z7M8 | Q0P670 |  |
|  | Q8NDB6 |  | Q5T7P8 | Q15238 | Q5VTH2 | Q86SI9 |  |
|  | A6NP61 |  | Q9NS28 | Q96A32 | Q7L273 | Q8N8X9 |  |
|  | P58215 |  | O15375 | Q9BQ95 | A2RUH7 | Q8NG41 |  |
|  | Q96D21 |  | Q7Z5H5 | A6NLC8 | Q15544 | Q9UFV1 |  |
|  | O95340 |  | Q8IX04 | O15479 | Q96GY0 | Q8IWE4 |  |
|  | Q70CQ4 |  | A8MZH6 |  | A6NN92 | O96013 |  |
|  | P17706 |  | P61247 |  | Q6ZU35 | Q6PIF2 |  |
|  | Q15390 |  | Q9HAY6 |  | Q5VVP1 | Q9Y3E7 |  |
|  | B0YJ81 |  | O95872 |  | Q9NPD3 | O75940 |  |
|  | O15374 |  | Q3SXM0 |  | Q7Z6L1 | Q6UWV6 |  |
|  | O95057 |  | O95206 |  | Q7Z304 | Q8N0U8 |  |
|  | Q96E66 |  | Q9H3Q1 |  | Q6ZMJ4 | P84085 |  |
|  | B2RXH2 |  | Q9BUP0 |  | P26885 | Q8NC44 |  |
|  | Q6NSJ0 |  | O94760 |  | Q86X51 | Q8NE00 |  |
|  | P25686 |  | Q9NUP9 |  | Q03135 | O95278 |  |
|  | Q8TA86 |  | Q96EF9 |  | Q9Y2J8 | Q03405 |  |
|  | Q9H0C1 |  | Q6AWC8 |  | Q1W4C9 | O75581 |  |
|  | B4E2M5 |  | P20042 |  | Q5TA78 | Q9H082 |  |
|  | Q9UNL4 |  | P15121 |  | Q14201 | Q9ULR0 |  |
|  | P08582 |  | A6NKL6 |  | Q58FF6 | A6NJT0 |  |
|  | Q5T1C6 |  | Q9P0M6 |  | Q9BYG8 | Q8WVF5 |  |
|  | Q15637 |  | Q96NT5 |  | P41595 | Q9H7B7 |  |
|  | O60346 |  | O60271 |  | Q8NGP4 | P22001 |  |
|  | P20618 |  | P51582 |  | Q96GX5 | Q9Y5A7 |  |
|  | Q00765 |  | Q86UN6 |  | Q8NEA4 | Q6IPW1 |  |
|  | Q6NYC1 |  | Q8N5I9 |  | Q8IYA7 | Q6NXE6 |  |
|  | Q7LDI9 |  | Q8N0W7 |  | Q9NYB0 | A6NNY8 |  |
|  | P49354 |  | Q8TCB7 |  | Q8N7X4 | P61565 |  |
|  | Q5JST6 |  | Q92630 |  | Q9HAB3 | P0DTF9 |  |
|  | P61073 |  | O00115 |  | A6NLE4 | Q9UBL9 |  |
|  | Q6PI98 |  | O00399 |  | A1A4Y4 | Q99811 |  |
|  | Q8NH74 |  | Q96HA8 |  | Q7L8S5 | Q8NAJ2 |  |
|  | P52298 |  | Q96A23 |  | P57081 | P41143 |  |
|  | Q9Y580 |  | Q6DWJ6 |  | Q8NGD3 | Q96IW7 |  |
|  | Q4G0A6 |  | Q9ULI2 |  | Q9UK13 | Q9GZM8 |  |
|  | P51884 |  | O43325 |  | Q13564 | P17213 |  |
|  | A0A075B6I7 | | P0DKB6 |  | Q86XF0 | P62487 |  |
|  | O94830 |  |  |  | P30405 | Q8TF47 |  |
|  | P09958 |  |  |  | P52564 | Q969H6 |  |
|  | Q5FYB0 |  |  |  | Q9HAE3 | O75530 |  |
|  | Q05925 |  |  |  | Q9UIJ5 | P48739 |  |
|  | Q92536 |  |  |  | P11684 | Q9BUI4 |  |
|  | Q9NY72 |  |  |  | P08253 | Q9BUK6 |  |
|  | A0A5F9ZHS7 | |  |  | Q14642 | Q9Y2D2 |  |
|  | P31944 |  |  |  | Q96GM8 | Q9UC06 |  |
|  | Q96EQ8 |  |  |  | Q86VF5 | A0A1B0GVG4 | |
|  | P0C7I6 |  |  |  | Q9NSB2 | Q86XP1 |  |
|  | Q9Y5K3 |  |  |  | A2RU30 | Q96S21 |  |
|  | Q8NFB2 |  |  |  | Q9H210 | Q86YL5 |  |
|  | Q9BRX8 |  |  |  | O14879 | Q9NNW7 |  |
|  | Q8N7X2 |  |  |  | Q9UHK0 | O60359 |  |
|  | Q96LR9 |  |  |  | Q9Y4K0 | Q8N8Z3 |  |
|  | A8MYV0 |  |  |  | Q6P1R3 | Q9Y2C3 |  |
|  | P01036 |  |  |  | A8MWD9 | P11117 |  |
|  | O95154 |  |  |  | Q8IYJ2 | O95478 |  |
|  | Q15831 |  |  |  | A0A075B6I0 | Q9Y2G1 |  |
|  | A0A1B0GUA9 | |  |  | P83859 | O95479 |  |
|  | P63128 |  |  |  | Q32NC0 | P42772 |  |
|  | O43709 |  |  |  | Q08493 | Q8NGK9 |  |
|  | Q6P1K2 |  |  |  | Q9H0X4 | Q2TAM9 |  |
|  | Q8NE31 |  |  |  | Q15846 | Q9UBR2 |  |
|  | Q16629 |  |  |  | Q5TA89 | O95678 |  |
|  | Q14451 |  |  |  | A6NIN4 | Q9HAP6 |  |
|  | Q52M93 |  |  |  | Q9H0A6 | Q9H3S4 |  |
|  | Q99626 |  |  |  | Q00444 | O75529 |  |
|  | Q9UJD0 |  |  |  | A0A286YF58 | P14784 |  |
|  | Q9Y232 |  |  |  | Q13495 | P12525 |  |
|  | Q9Y644 |  |  |  | Q9H2S6 | Q96HY7 |  |
|  | P51812 |  |  |  | Q8N344 | Q13153 |  |
|  | Q2M3A8 |  |  |  | Q9GZQ8 | Q9BZ98 |  |
|  | Q8NGJ1 |  |  |  | O43174 | Q13409 |  |
|  | P61960 |  |  |  | Q5VZ52 | P20309 |  |
|  | Q5R3F8 |  |  |  | Q9HDB5 | Q96LK0 |  |
|  | Q9BZ23 |  |  |  | Q9BQ16 | P35070 |  |
|  | P22695 |  |  |  | Q9Y397 | P52597 |  |
|  | Q14145 |  |  |  | P57775 | Q96EX3 |  |
|  | Q9BT49 |  |  |  | Q96D21 | P03956 |  |
|  | O43681 |  |  |  | O95674 | P15813 |  |
|  | Q9BYH1 |  |  |  | Q9Y221 | Q5VT66 |  |
|  | Q7Z5L9 |  |  |  | Q9UPM6 | Q6P1Q0 |  |
|  | Q96R67 |  |  |  | O95867 | Q86WC4 |  |
|  | Q13526 |  |  |  | Q8TBF2 | O95822 |  |
|  | Q9NSC5 |  |  |  | O15321 | Q5JRS4 |  |
|  | P07477 |  |  |  | P00492 | A1L190 |  |
|  | P53634 |  |  |  | Q6ZMT1 | Q5T681 |  |
|  | O43657 |  |  |  | Q7Z5H4 | P10828 |  |
|  | O43688 |  |  |  | O95057 | Q93091 |  |
|  | O75600 |  |  |  | Q6UXB8 | Q14469 |  |
|  | O43175 |  |  |  | B2RXH2 | Q569G3 |  |
|  | Q9NXR8 |  |  |  | Q9H0C1 | A5PLL7 |  |
|  | P12273 |  |  |  | P55008 | Q96AP7 |  |
|  | Q08722 |  |  |  | Q8N2Z9 | Q7Z3C6 |  |
|  | Q5VZV1 |  |  |  | P0DJH9 | O15213 |  |
|  | O75695 |  |  |  | Q9Y227 | Q96CS3 |  |
|  | P09960 |  |  |  | Q6ZNG2 | Q7Z7M1 |  |
|  | P59046 |  |  |  | Q8N6L0 | P20592 |  |
|  | Q9HBA0 |  |  |  | Q9BQP9 | P24534 |  |
|  | P56915 |  |  |  | P08582 | A0A1B0GUS0 | |
|  | Q9GZM5 |  |  |  | Q8WUF8 | Q15617 |  |
|  | Q7Z5D8 |  |  |  | O75462 | P0DV77 |  |
|  | P0DPQ3 |  |  |  | Q9BZL3 | O43829 |  |
|  | Q9UBE8 |  |  |  | P17787 | Q9BTK6 |  |
|  | P22748 |  |  |  | Q9HB03 | Q9Y337 |  |
|  | Q8N4P6 |  |  |  | Q15759 | Q8N888 |  |
|  | A6NMZ2 |  |  |  | Q9P283 | P17844 |  |
|  | Q01726 |  |  |  | Q15291 | P49748 |  |
|  | P35790 |  |  |  | Q14990 | O60704 |  |
|  | Q8IYK8 |  |  |  | Q99574 | Q9BU89 |  |
|  | Q96D59 |  |  |  | Q86TL0 | Q9NNX1 |  |
|  | P01303 |  |  |  | Q7Z2E3 | Q96QH2 |  |
|  | Q9UPV7 |  |  |  | Q6ZSZ6 | Q9H9A5 |  |
|  | P61006 |  |  |  | Q92481 | Q8IYG6 |  |
|  | Q9H4P4 |  |  |  | Q9NP08 | Q9HD26 |  |
|  | Q06710 |  |  |  | Q9NVS2 | Q8IVP9 |  |
|  | Q53S58 |  |  |  | P30542 | P34741 |  |
|  | Q7Z3D4 |  |  |  | O15541 | O76013 |  |
|  | P0C7U3 |  |  |  | P21730 | Q99755 |  |
|  | P54762 |  |  |  | P51116 | Q8IZL2 |  |
|  | Q16655 |  |  |  | O75781 | Q9NZZ3 |  |
|  | Q9UQM7 |  |  |  | Q92536 | A4D2B8 |  |
|  | Q7Z434 |  |  |  | A0A5F9ZHS7 | Q08ER8 |  |
|  | P0DPE3 |  |  |  | P34903 | P08887 |  |
|  | Q5JTV8 |  |  |  | P31944 | O75170 |  |
|  | Q9BXS5 |  |  |  | Q7Z4W1 | O60688 |  |
|  | P16455 |  |  |  | Q96EQ8 | P61201 |  |
|  | P22460 |  |  |  | P05154 | Q96EG1 |  |
|  | O75953 |  |  |  | Q5T6M2 | Q9Y5X1 |  |
|  | Q6NTE8 |  |  |  | Q9BRX8 | P15259 |  |
|  | Q02930 |  |  |  | O15063 | Q6ICB4 |  |
|  | Q92886 |  |  |  | P36542 | O00458 |  |
|  | Q96LT4 |  |  |  | Q96NG8 | O15516 |  |
|  | Q9H840 |  |  |  | Q9Y2I6 | Q2VWA4 |  |
|  | Q6IQ20 |  |  |  | O14791 | Q6IF99 |  |
|  | Q9C0H6 |  |  |  | Q96K30 | P41222 |  |
|  | Q6UXY1 |  |  |  | Q96LR9 | C9J1S8 |  |
|  | Q9HB07 |  |  |  | P43351 | Q9BXA6 |  |
|  | Q8N752 |  |  |  | Q8NGS6 | Q96DG6 |  |
|  | Q86XK2 |  |  |  | Q15831 | Q9Y6S9 |  |
|  | Q9NZP2 |  |  |  | Q6F5E7 | Q9Y5F1 |  |
|  | Q16670 |  |  |  | P43365 | A0A1B0GVG6 | |
|  | Q9Y2C2 |  |  |  | Q9UHA3 | O95858 |  |
|  | O75677 |  |  |  | O43709 | Q15842 |  |
|  | Q8IX15 |  |  |  | Q6UW01 | Q96P70 |  |
|  | Q5BKX5 |  |  |  | Q9Y672 | Q6P444 |  |
|  | Q9BWW7 | |  |  | Q96KN8 | Q8TF21 |  |
|  | O94855 |  |  |  | Q9Y6I7 | Q01813 |  |
|  | Q5JXM2 |  |  |  | Q8IX29 | Q9Y6F8 |  |
|  | Q8NGH3 |  |  |  | Q9H158 | Q16877 |  |
|  | Q6UWL6 |  |  |  | Q99626 | P22692 |  |
|  | P23528 |  |  |  | P49419 | Q13332 |  |
|  | O43516 |  |  |  | P28562 | P62906 |  |
|  | Q8WU10 |  |  |  | Q86X60 | Q8IXX5 |  |
|  | Q7RTT6 |  |  |  | A0A1B0GUX0 | Q96KP1 |  |
|  | P41208 |  |  |  | P08246 | Q3ZAQ7 |  |
|  | Q9Y2T3 |  |  |  | Q9NX40 | Q5GH76 |  |
|  | Q9BZV2 |  |  |  | Q8NGJ1 | Q8NE79 |  |
|  | Q8WV48 |  |  |  | Q99986 | A0A1B0GVX0 | |
|  | P08567 |  |  |  | Q96M66 | Q96EL3 |  |
|  | Q9BUE0 |  |  |  | Q12800 | Q9P127 |  |
|  | Q00169 |  |  |  | Q6PD74 | Q9BXW4 |  |
|  | Q8TBY0 |  |  |  | Q5R3F8 | Q9NWM3 | |
|  | P29120 |  |  |  | Q9BXW7 | Q5VVY1 |  |
|  | O95881 |  |  |  | Q96N03 | O76099 |  |
|  | Q8NH02 |  |  |  | Q13303 | Q6PIS1 |  |
|  | O15269 |  |  |  | Q13634 | Q9UK85 |  |
|  | Q17RF5 |  |  |  | Q5SZJ8 | Q92519 |  |
|  | O95848 |  |  |  | A6NI79 | Q13547 |  |
|  | Q8NI28 |  |  |  | P55040 | Q9NP74 |  |
|  | Q96PK6 |  |  |  | P50440 | Q6ZTW0 |  |
|  | Q5VT03 |  |  |  | Q8NFW5 | Q8TAA1 |  |
|  |  |  |  |  | P11831 | Q9HAJ7 |  |
|  |  |  |  |  | Q05048 | A0A1B0GUI7 | |
|  |  |  |  |  | Q96JP9 | Q5JXB2 |  |
|  |  |  |  |  | P29034 | Q66K64 |  |
|  |  |  |  |  | Q9UNX3 | A6NIJ9 |  |
|  |  |  |  |  | Q9H3Y6 | Q8NFD4 |  |
|  |  |  |  |  | O43688 | Q9H1A7 |  |
|  |  |  |  |  | Q9NUG6 | O15554 |  |
|  |  |  |  |  | O76075 | Q14094 |  |
|  |  |  |  |  | P43304 | Q02094 |  |
|  |  |  |  |  | O75521 | Q9UKY4 |  |
|  |  |  |  |  | P10073 | O75838 |  |
|  |  |  |  |  | Q5T124 | P05543 |  |
|  |  |  |  |  | Q7Z6P3 | Q5GAN3 |  |
|  |  |  |  |  | P10109 | Q96IW2 |  |
|  |  |  |  |  | P56915 | Q92629 |  |
|  |  |  |  |  | O43399 | Q53QW1 |  |
|  |  |  |  |  | Q86UD4 | Q9GZX7 |  |
|  |  |  |  |  | Q92917 | A6NIU2 |  |
|  |  |  |  |  | Q9BQB6 | O60641 |  |
|  |  |  |  |  | A1L167 | Q9Y4A0 |  |
|  |  |  |  |  | Q92570 | O75204 |  |
|  |  |  |  |  | Q9UK08 | Q49AN0 |  |
|  |  |  |  |  | Q9ULR3 | Q14681 |  |
|  |  |  |  |  | Q5D0E6 | Q12792 |  |
|  |  |  |  |  | P13805 | Q8TAV4 |  |
|  |  |  |  |  | P29972 | Q9Y388 |  |
|  |  |  |  |  | P35557 | P60896 |  |
|  |  |  |  |  | Q9NP72 | O95405 |  |
|  |  |  |  |  | Q03938 | Q5VWJ9 |  |
|  |  |  |  |  | P35790 | Q8WV93 |  |
|  |  |  |  |  | Q09028 | Q9Y6E0 |  |
|  |  |  |  |  | P01303 | O76070 |  |
|  |  |  |  |  | P61006 | Q6RFH5 |  |
|  |  |  |  |  | Q6PI78 | Q8NHC4 |  |
|  |  |  |  |  | Q06710 | Q96HA1 |  |
|  |  |  |  |  | Q5T7P8 | Q8NFI4 |  |
|  |  |  |  |  | Q9NS28 | Q9C0K0 |  |
|  |  |  |  |  | Q7Z3D4 | Q9NRC8 |  |
|  |  |  |  |  | O15375 | P55160 |  |
|  |  |  |  |  | Q7Z5H5 | P52945 |  |
|  |  |  |  |  | P18283 | Q9H6T0 |  |
|  |  |  |  |  | Q9BUV0 | C0HM02 |  |
|  |  |  |  |  | Q9HCL3 | Q8N814 |  |
|  |  |  |  |  | O95872 | Q86VY9 |  |
|  |  |  |  |  | Q07666 | Q92963 |  |
|  |  |  |  |  | Q14213 | Q9BY15 |  |
|  |  |  |  |  | Q9HCL0 | Q8N9V6 |  |
|  |  |  |  |  | Q5VVH5 | A0A1B0GVQ3 | |
|  |  |  |  |  | Q13951 | Q96IR7 |  |
|  |  |  |  |  | Q3SXM0 | A0MZ66 |  |
|  |  |  |  |  | Q5JTV8 | Q8WU03 |  |
|  |  |  |  |  | P62316 | Q8WUB8 |  |
|  |  |  |  |  | Q9BUP0 | Q9HB89 |  |
|  |  |  |  |  | Q5VWW1 | A0A1W2PR19 | |
|  |  |  |  |  | O94760 | P40925 |  |
|  |  |  |  |  | Q9BV44 | Q9UNA1 |  |
|  |  |  |  |  | Q6NZY7 | Q8N567 |  |
|  |  |  |  |  | Q9H6J7 | P33992 |  |
|  |  |  |  |  | Q9NUP9 | P15248 |  |
|  |  |  |  |  | A0A1W2PPL8 | E9PB15 |  |
|  |  |  |  |  | Q96EF9 | Q86T29 |  |
|  |  |  |  |  | Q13352 | Q9GZS0 |  |
|  |  |  |  |  | Q6ZMU5 | Q86SQ9 |  |
|  |  |  |  |  | P48426 | Q8N123 |  |
|  |  |  |  |  | Q6AWC8 | Q9HAS0 |  |
|  |  |  |  |  | Q96LT4 | Q9Y225 |  |
|  |  |  |  |  | Q6IQ20 | P07093 |  |
|  |  |  |  |  | Q8N752 | P52429 |  |
|  |  |  |  |  | Q9H6D8 | Q6IN84 |  |
|  |  |  |  |  | P15121 | Q8NG35 |  |
|  |  |  |  |  | Q9H0N5 | Q8N9U9 |  |
|  |  |  |  |  | Q16670 | Q8WXF3 |  |
|  |  |  |  |  | A6NKL6 | Q9BR76 |  |
|  |  |  |  |  | P04629 | Q9P215 |  |
|  |  |  |  |  | Q13938 | Q9UNH6 |  |
|  |  |  |  |  | Q5T089 | A2RUC4 |  |
|  |  |  |  |  | Q9Y5E6 | P0DKX0 |  |
|  |  |  |  |  | O60384 | P82933 |  |
|  |  |  |  |  | Q9HD34 | Q96PN8 |  |
|  |  |  |  |  | Q96NT5 | Q9GZY4 |  |
|  |  |  |  |  | O95810 | O76015 |  |
|  |  |  |  |  | Q5BKX5 | P55039 |  |
|  |  |  |  |  | Q8N5I9 | Q658T7 |  |
|  |  |  |  |  | Q9Y240 | Q8IU54 |  |
|  |  |  |  |  | Q8N0W7 | Q8NCE0 |  |
|  |  |  |  |  | Q92630 | Q92537 |  |
|  |  |  |  |  | Q99853 | Q8N743 |  |
|  |  |  |  |  | O00399 | Q14512 |  |
|  |  |  |  |  | Q8WUD4 | Q9UHC3 |  |
|  |  |  |  |  | Q96A23 | Q2MJR0 |  |
|  |  |  |  |  | A8MV23 | P02794 |  |
|  |  |  |  |  | O43325 | Q8NHY2 |  |
|  |  |  |  |  | A4D256 | Q14129 |  |
|  |  |  |  |  | Q53GA4 | P59282 |  |
|  |  |  |  |  | Q9UHC7 | Q5VZF2 |  |
|  |  |  |  |  | Q8TBC5 | P08311 |  |
|  |  |  |  |  | O95881 | Q7Z5U6 |  |
|  |  |  |  |  | P59103 | Q9BYG4 |  |
|  |  |  |  |  | Q15238 | Q8WUU4 |  |
|  |  |  |  |  | P0DKB6 | Q9NWX6 |  |
|  |  |  |  |  | Q17RF5 | Q9Y271 |  |
|  |  |  |  |  | Q9BQ95 | Q16816 |  |
|  |  |  |  |  | A6NLC8 | Q9Y4H2 |  |
|  |  |  |  |  | O15479 | Q8N7B1 |  |
|  |  |  |  |  |  | Q96RD7 |  |
|  |  |  |  |  |  | P98170 |  |
|  |  |  |  |  |  | Q9Y4X0 |  |
|  |  |  |  |  |  | P82932 |  |
|  |  |  |  |  |  | Q7RTY3 |  |
|  |  |  |  |  |  | Q86WN1 |  |
|  |  |  |  |  |  | Q8NG98 |  |
|  |  |  |  |  |  | O60565 |  |
|  |  |  |  |  |  | Q8NCK3 |  |
|  |  |  |  |  |  | Q9H074 |  |
|  |  |  |  |  |  | Q9UBK8 |  |
|  |  |  |  |  |  | P10909 |  |
|  |  |  |  |  |  | Q96BK5 |  |
|  |  |  |  |  |  | P67870 |  |
|  |  |  |  |  |  | Q8NEP7 |  |
|  |  |  |  |  |  | Q49AR2 |  |
|  |  |  |  |  |  | Q96MF7 |  |
|  |  |  |  |  |  | Q9P1U0 |  |
|  |  |  |  |  |  | Q5VT33 |  |
|  |  |  |  |  |  | Q6UW49 |  |
|  |  |  |  |  |  | Q9H106 |  |
|  |  |  |  |  |  | P36915 |  |
|  |  |  |  |  |  | Q02575 |  |
|  |  |  |  |  |  | Q96CT7 |  |
|  |  |  |  |  |  | P19525 |  |
|  |  |  |  |  |  | Q5SV17 |  |
|  |  |  |  |  |  | O60299 |  |
|  |  |  |  |  |  | Q13515 |  |
|  |  |  |  |  |  | Q6N063 |  |
|  |  |  |  |  |  | P0CG00 |  |
|  |  |  |  |  |  | Q9Y5G0 |  |
|  |  |  |  |  |  | Q86XP0 |  |
|  |  |  |  |  |  | Q8N6Q8 |  |
|  |  |  |  |  |  | Q96EW2 |  |
|  |  |  |  |  |  | Q7L8J4 |  |
|  |  |  |  |  |  | Q5VYX0 |  |
|  |  |  |  |  |  | P57768 |  |
|  |  |  |  |  |  | P78330 |  |
|  |  |  |  |  |  | Q96J92 |  |
|  |  |  |  |  |  | O95256 |  |
|  |  |  |  |  |  | Q6PJQ5 |  |
|  |  |  |  |  |  | Q6P2H3 |  |
|  |  |  |  |  |  | Q86Z02 |  |
|  |  |  |  |  |  | Q9NWA0 |  |
|  |  |  |  |  |  | Q6ZUL3 |  |
|  |  |  |  |  |  | P59095 |  |
|  |  |  |  |  |  | A6NGU7 |  |
|  |  |  |  |  |  | O95863 |  |
|  |  |  |  |  |  | Q9H8S9 |  |
|  |  |  |  |  |  | A6NHQ4 |  |
|  |  |  |  |  |  | Q8NA92 |  |
|  |  |  |  |  |  | Q9UF02 |  |
|  |  |  |  |  |  | P35227 |  |
|  |  |  |  |  |  | Q96C92 |  |
|  |  |  |  |  |  | Q8N3C7 |  |
|  |  |  |  |  |  | Q9P0J1 |  |
|  |  |  |  |  |  | A7MD48 |  |
|  |  |  |  |  |  | Q9HAC8 |  |
|  |  |  |  |  |  | Q9UJX3 |  |
|  |  |  |  |  |  | O00217 |  |
|  |  |  |  |  |  | P11940 |  |
|  |  |  |  |  |  | O60701 |  |
|  |  |  |  |  |  | Q6ZRR5 |  |
|  |  |  |  |  |  | Q86WV6 |  |
|  |  |  |  |  |  | Q8N7R7 |  |
|  |  |  |  |  |  | P08697 |  |
|  |  |  |  |  |  | Q9UHY7 |  |
|  |  |  |  |  |  | Q7Z418 |  |
|  |  |  |  |  |  | A0A1B0GUS4 | |
|  |  |  |  |  |  | Q86UD7 |  |
|  |  |  |  |  |  | Q3SY05 |  |
|  |  |  |  |  |  | O60942 |  |
|  |  |  |  |  |  | A6NP61 |  |
|  |  |  |  |  |  | P49716 |  |
|  |  |  |  |  |  | Q96L46 |  |
|  |  |  |  |  |  | P15391 |  |
|  |  |  |  |  |  | Q16656 |  |
|  |  |  |  |  |  | Q8NE28 |  |
|  |  |  |  |  |  | O95340 |  |
|  |  |  |  |  |  | Q96BW5 |  |
|  |  |  |  |  |  | C9JR72 |  |
|  |  |  |  |  |  | Q9NS73 |  |
|  |  |  |  |  |  | P17706 |  |
|  |  |  |  |  |  | O95936 |  |
|  |  |  |  |  |  | P31941 |  |
|  |  |  |  |  |  | Q15390 |  |
|  |  |  |  |  |  | Q6ZSA7 |  |
|  |  |  |  |  |  | B0YJ81 |  |
|  |  |  |  |  |  | O15260 |  |
|  |  |  |  |  |  | Q63HQ0 |  |
|  |  |  |  |  |  | A8MXE2 |  |
|  |  |  |  |  |  | P26038 |  |
|  |  |  |  |  |  | Q5U651 |  |
|  |  |  |  |  |  | O15374 |  |
|  |  |  |  |  |  | Q9Y5E3 |  |
|  |  |  |  |  |  | P52757 |  |
|  |  |  |  |  |  | Q6UXH8 |  |
|  |  |  |  |  |  | Q96E66 |  |
|  |  |  |  |  |  | O00478 |  |
|  |  |  |  |  |  | P13995 |  |
|  |  |  |  |  |  | P25686 |  |
|  |  |  |  |  |  | Q96PB7 |  |
|  |  |  |  |  |  | Q15382 |  |
|  |  |  |  |  |  | Q8NFK1 |  |
|  |  |  |  |  |  | Q12934 |  |
|  |  |  |  |  |  | Q8IV35 |  |
|  |  |  |  |  |  | Q8TDN6 |  |
|  |  |  |  |  |  | Q9H3M9 |  |
|  |  |  |  |  |  | O75631 |  |
|  |  |  |  |  |  | Q96DE9 |  |
|  |  |  |  |  |  | P49757 |  |
|  |  |  |  |  |  | Q9Y2E6 |  |
|  |  |  |  |  |  | P01127 |  |
|  |  |  |  |  |  | C9J442 |  |
|  |  |  |  |  |  | Q9BUG6 |  |
|  |  |  |  |  |  | Q9BXM9 |  |
|  |  |  |  |  |  | Q9P086 |  |
|  |  |  |  |  |  | P60880 |  |
|  |  |  |  |  |  | Q9Y2T2 |  |
|  |  |  |  |  |  | O75818 |  |
|  |  |  |  |  |  | Q9NQ94 |  |
|  |  |  |  |  |  | Q8N4U5 |  |
|  |  |  |  |  |  | P20618 |  |
|  |  |  |  |  |  | Q6NYC1 |  |
|  |  |  |  |  |  | Q9H560 |  |
|  |  |  |  |  |  | Q9NX62 |  |
|  |  |  |  |  |  | Q9UHE5 |  |
|  |  |  |  |  |  | P52306 |  |
|  |  |  |  |  |  | P61073 |  |
|  |  |  |  |  |  | Q8NH74 |  |
|  |  |  |  |  |  | Q9Y543 |  |
|  |  |  |  |  |  | A0JD36 |  |
|  |  |  |  |  |  | O43598 |  |
|  |  |  |  |  |  | P52298 |  |
|  |  |  |  |  |  | Q9Y580 |  |
|  |  |  |  |  |  | A2A288 |  |
|  |  |  |  |  |  | O95948 |  |
|  |  |  |  |  |  | A8MSI8 |  |
|  |  |  |  |  |  | P09958 |  |
|  |  |  |  |  |  | Q8TDR2 |  |
|  |  |  |  |  |  | Q86V24 |  |
|  |  |  |  |  |  | O15392 |  |
|  |  |  |  |  |  | Q05925 |  |
|  |  |  |  |  |  | Q9NY72 |  |
|  |  |  |  |  |  | Q9UFG5 |  |
|  |  |  |  |  |  | A6NHZ5 |  |
|  |  |  |  |  |  | P62857 |  |
|  |  |  |  |  |  | Q5JTZ9 |  |
|  |  |  |  |  |  | Q8NGD1 |  |
|  |  |  |  |  |  | Q9NXR7 |  |
|  |  |  |  |  |  | P34896 |  |
|  |  |  |  |  |  | P0C7I6 |  |
|  |  |  |  |  |  | Q96HJ9 |  |
|  |  |  |  |  |  | Q9Y5K3 |  |
|  |  |  |  |  |  | Q8NFB2 |  |
|  |  |  |  |  |  | P15976 |  |
|  |  |  |  |  |  | Q8IX05 |  |
|  |  |  |  |  |  | P02760 |  |
|  |  |  |  |  |  | Q9HCK0 |  |
|  |  |  |  |  |  | Q6IQ16 |  |
|  |  |  |  |  |  | Q7RTX9 |  |
|  |  |  |  |  |  | Q969P0 |  |
|  |  |  |  |  |  | P01036 |  |
|  |  |  |  |  |  | O94900 |  |
|  |  |  |  |  |  | O95154 |  |
|  |  |  |  |  |  | Q6NUT3 |  |
|  |  |  |  |  |  | Q9BPU9 |  |
|  |  |  |  |  |  | Q69YN2 |  |
|  |  |  |  |  |  | Q16629 |  |
|  |  |  |  |  |  | Q9NZU5 |  |
|  |  |  |  |  |  | P04844 |  |
|  |  |  |  |  |  | Q9UJD0 |  |
|  |  |  |  |  |  | Q9Y232 |  |
|  |  |  |  |  |  | Q9NQ75 |  |
|  |  |  |  |  |  | P17483 |  |
|  |  |  |  |  |  | P51812 |  |
|  |  |  |  |  |  | Q96JB8 |  |
|  |  |  |  |  |  | Q2M3A8 |  |
|  |  |  |  |  |  | Q8NH60 |  |
|  |  |  |  |  |  | Q96P88 |  |
|  |  |  |  |  |  | Q9NS93 |  |
|  |  |  |  |  |  | P61960 |  |
|  |  |  |  |  |  | Q9BZ23 |  |
|  |  |  |  |  |  | Q9Y625 |  |
|  |  |  |  |  |  | P22695 |  |
|  |  |  |  |  |  | Q3MIS6 |  |
|  |  |  |  |  |  | Q9BUM1 |  |
|  |  |  |  |  |  | Q9NX36 |  |
|  |  |  |  |  |  | Q96M63 |  |
|  |  |  |  |  |  | Q14145 |  |
|  |  |  |  |  |  | O43681 |  |
|  |  |  |  |  |  | P38646 |  |
|  |  |  |  |  |  | P38606 |  |
|  |  |  |  |  |  | Q93038 |  |
|  |  |  |  |  |  | O43699 |  |
|  |  |  |  |  |  | Q5JT25 |  |
|  |  |  |  |  |  | Q9BYH1 |  |
|  |  |  |  |  |  | A0A1B0GUJ8 | |
|  |  |  |  |  |  | Q9BT56 |  |
|  |  |  |  |  |  | O60911 |  |
|  |  |  |  |  |  | Q96R67 |  |
|  |  |  |  |  |  | A8K7I4 |  |
|  |  |  |  |  |  | Q96IS3 |  |
|  |  |  |  |  |  | P53634 |  |
|  |  |  |  |  |  | O43657 |  |
|  |  |  |  |  |  | Q8NI37 |  |
|  |  |  |  |  |  | O43175 |  |
|  |  |  |  |  |  | Q9NXR8 |  |
|  |  |  |  |  |  | A8MVW0 |  |
|  |  |  |  |  |  | P12273 |  |
|  |  |  |  |  |  | Q8WZ71 |  |
|  |  |  |  |  |  | P28072 |  |
|  |  |  |  |  |  | Q8IXF9 |  |
|  |  |  |  |  |  | O75106 |  |
|  |  |  |  |  |  | Q5VZM2 |  |
|  |  |  |  |  |  | P14317 |  |
|  |  |  |  |  |  | Q8NA75 |  |
|  |  |  |  |  |  | Q9H063 |  |
|  |  |  |  |  |  | Q9H5K3 |  |
|  |  |  |  |  |  | A6NC05 |  |
|  |  |  |  |  |  | O75884 |  |
|  |  |  |  |  |  | O95136 |  |
|  |  |  |  |  |  | P0DPQ3 |  |
|  |  |  |  |  |  | P49759 |  |
|  |  |  |  |  |  | Q9UBE8 |  |
|  |  |  |  |  |  | P22748 |  |
|  |  |  |  |  |  | P57727 |  |
|  |  |  |  |  |  | Q8N4P6 |  |
|  |  |  |  |  |  | Q01726 |  |
|  |  |  |  |  |  | P04075 |  |
|  |  |  |  |  |  | Q13227 |  |
|  |  |  |  |  |  | O95865 |  |
|  |  |  |  |  |  | Q9GZP7 |  |
|  |  |  |  |  |  | Q9Y3E0 |  |
|  |  |  |  |  |  | Q5TFG8 |  |
|  |  |  |  |  |  | Q8N7Q3 |  |
|  |  |  |  |  |  | Q96D59 |  |
|  |  |  |  |  |  | Q9H773 |  |
|  |  |  |  |  |  | Q13093 |  |
|  |  |  |  |  |  | Q6UN15 |  |
|  |  |  |  |  |  | Q86W34 |  |
|  |  |  |  |  |  | Q9NZ72 |  |
|  |  |  |  |  |  | Q9UPV7 |  |
|  |  |  |  |  |  | O60894 |  |
|  |  |  |  |  |  | Q9H4P4 |  |
|  |  |  |  |  |  | Q5BJD5 |  |
|  |  |  |  |  |  | Q6PK18 |  |
|  |  |  |  |  |  | Q9P299 |  |
|  |  |  |  |  |  | Q8NAP8 |  |
|  |  |  |  |  |  | Q96PB8 |  |
|  |  |  |  |  |  | Q8N9L1 |  |
|  |  |  |  |  |  | Q8NH80 |  |
|  |  |  |  |  |  | A6NJI1 |  |
|  |  |  |  |  |  | P13473 |  |
|  |  |  |  |  |  | Q8IX04 |  |
|  |  |  |  |  |  | Q9NX76 |  |
|  |  |  |  |  |  | Q9P2J9 |  |
|  |  |  |  |  |  | P05783 |  |
|  |  |  |  |  |  | Q9NTU4 |  |
|  |  |  |  |  |  | A8MZH6 |  |
|  |  |  |  |  |  | P61247 |  |
|  |  |  |  |  |  | Q8WXF1 |  |
|  |  |  |  |  |  | Q16655 |  |
|  |  |  |  |  |  | Q9HAY6 |  |
|  |  |  |  |  |  | Q8IYT1 |  |
|  |  |  |  |  |  | Q629K1 |  |
|  |  |  |  |  |  | Q9BSN7 |  |
|  |  |  |  |  |  | P27815 |  |
|  |  |  |  |  |  | A8MW95 |  |
|  |  |  |  |  |  | Q9HCY8 |  |
|  |  |  |  |  |  | Q7Z4H4 |  |
|  |  |  |  |  |  | Q9UL16 |  |
|  |  |  |  |  |  | O95206 |  |
|  |  |  |  |  |  | P36222 |  |
|  |  |  |  |  |  | Q5W0B1 |  |
|  |  |  |  |  |  | P16455 |  |
|  |  |  |  |  |  | P22460 |  |
|  |  |  |  |  |  | P41227 |  |
|  |  |  |  |  |  | Q9HBL6 |  |
|  |  |  |  |  |  | A0A1B0GTH6 | |
|  |  |  |  |  |  | A6NDH6 |  |
|  |  |  |  |  |  | Q6NTE8 |  |
|  |  |  |  |  |  | Q9HC23 |  |
|  |  |  |  |  |  | Q02930 |  |
|  |  |  |  |  |  | Q8WWY6 | |
|  |  |  |  |  |  | Q92886 |  |
|  |  |  |  |  |  | P20042 |  |
|  |  |  |  |  |  | Q9NQW6 | |
|  |  |  |  |  |  | Q86XK2 |  |
|  |  |  |  |  |  | Q9NZP2 |  |
|  |  |  |  |  |  | Q9HCM9 |  |
|  |  |  |  |  |  | Q17RB8 |  |
|  |  |  |  |  |  | Q32MZ4 |  |
|  |  |  |  |  |  | P05112 |  |
|  |  |  |  |  |  | P48051 |  |
|  |  |  |  |  |  | O43854 |  |
|  |  |  |  |  |  | Q9P0M6 |  |
|  |  |  |  |  |  | O00180 |  |
|  |  |  |  |  |  | Q9NQZ5 |  |
|  |  |  |  |  |  | Q8IY67 |  |
|  |  |  |  |  |  | O75677 |  |
|  |  |  |  |  |  | Q15014 |  |
|  |  |  |  |  |  | Q3KRA9 |  |
|  |  |  |  |  |  | P51582 |  |
|  |  |  |  |  |  | Q86UN6 |  |
|  |  |  |  |  |  | Q9H7B2 |  |
|  |  |  |  |  |  | Q8WXF5 |  |
|  |  |  |  |  |  | Q86UU0 |  |
|  |  |  |  |  |  | P22304 |  |
|  |  |  |  |  |  | P07204 |  |
|  |  |  |  |  |  | Q5T0U0 |  |
|  |  |  |  |  |  | Q5JXM2 |  |
|  |  |  |  |  |  | Q8NGH3 |  |
|  |  |  |  |  |  | Q8TCB7 |  |
|  |  |  |  |  |  | Q96PC3 |  |
|  |  |  |  |  |  | Q9NX18 |  |
|  |  |  |  |  |  | Q6P9G9 |  |
|  |  |  |  |  |  | Q14093 |  |
|  |  |  |  |  |  | O00115 |  |
|  |  |  |  |  |  | Q96HA8 |  |
|  |  |  |  |  |  | O43516 |  |
|  |  |  |  |  |  | Q6DWJ6 |  |
|  |  |  |  |  |  | Q8N183 |  |
|  |  |  |  |  |  | P37287 |  |
|  |  |  |  |  |  | Q8WV48 |  |
|  |  |  |  |  |  | Q9ULI2 |  |
|  |  |  |  |  |  | Q9BUE0 |  |
|  |  |  |  |  |  | A5YM72 |  |
|  |  |  |  |  |  | Q8WUJ1 |  |
|  |  |  |  |  |  | Q9NRP4 |  |
|  |  |  |  |  |  | O43869 |  |
|  |  |  |  |  |  | Q9Y3M2 |  |
|  |  |  |  |  |  | Q8TBY0 |  |
|  |  |  |  |  |  | P29120 |  |
|  |  |  |  |  |  | Q8NGS0 |  |
|  |  |  |  |  |  | Q5PSV4 |  |
|  |  |  |  |  |  | O43307 |  |
|  |  |  |  |  |  | P31995 |  |
|  |  |  |  |  |  | Q8NGF8 |  |
|  |  |  |  |  |  | Q96A32 |  |
|  |  |  |  |  |  | O75795 |  |
|  |  |  |  |  |  | Q8IYI0 |  |
|  |  |  |  |  |  | Q8NI28 |  |
|  |  |  |  |  |  | Q96PK6 |  |
|  |  |  |  |  |  | Q14397 |  |
