# Supplementary material for: Proteomic profiling of urinary extracellular vesicles differentiates breast cancer patients from healthy women
Source: PLoS One. 2023 Nov 3;18(11):e0291574. doi: 10.1371/journal.pone.0291574 (PMC10624262; doi:10.1371/journal.pone.0291574)
Supplement: S1 Raw image — (PDF) [file pone.0291574.s011.pdf]

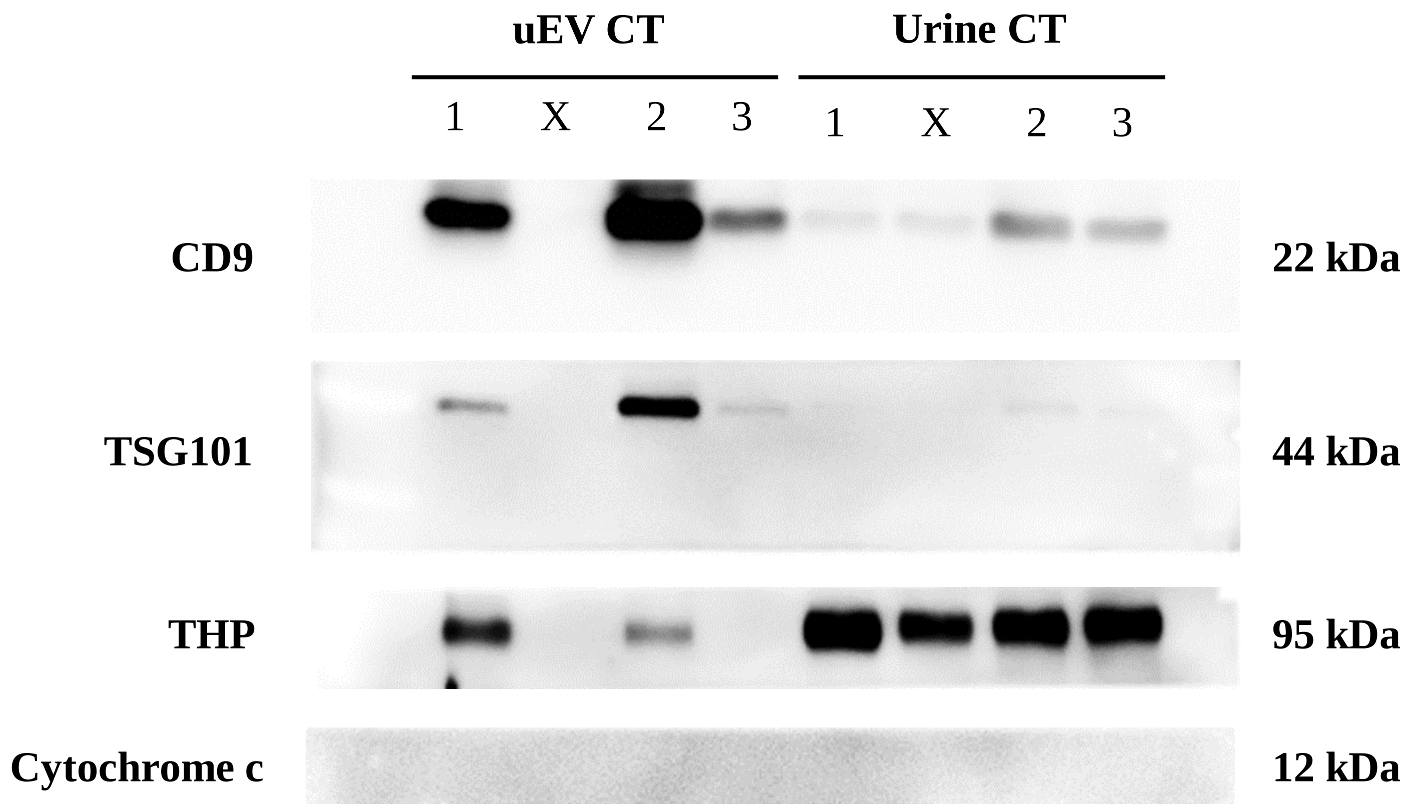

S1_raw_image: The original blot image used to generate Fig. 3A Characterization of uEVs, Each membrane was visualized and captured through a chemiluminescence imager (Alliance Q9 Advanced, UVITEC) to detect the specific protein bands. The samples were matched uEV pellets and urine from individuals in the healthy control (CT) group (n = 4). The lane was not included in the final figure, as indicated by a "X" above the lane on the original blot.
